# HPLC-DAD and UHPLC/QTOF-MS Analysis of Polyphenols in Extracts of the African Species *Combretum* *padoides*, *C. zeyheri* and *C. psidioides* Related to Their Antimycobacterial Activity

**DOI:** 10.3390/antibiotics9080459

**Published:** 2020-07-29

**Authors:** Pia Fyhrquist, Enass Y. A. Salih, Satu Helenius, Into Laakso, Riitta Julkunen-Tiitto

**Affiliations:** 1 Faculty of Pharmacy, Division of Pharmaceutical Biosciences, Viikki Biocenter, P.O. Box 56, FIN-00014, University of Helsinki, 00100 Helsinki, Finland; enass.salih@helsinki.fi (E.Y.A.S.); skhelenius@gmail.com (S.H.); into.laakso@helsinki.fi (I.L.); 2 Department of Forest Products and Industries, Shambat Campus, SUD-13314, University of Khartoum, Khartoum 11111, Sudan; 3 Faculty of Science and Forestry, Department of Environmental and Biological Sciences, University of Eastern Finland, 80101 Joensuu, Finland; rjt@uef.fi

**Keywords:** *Combretum* spp., antimycobacterial, hydrolysable tannins, stilbenes, African traditional medicine

## Abstract

*Combretum padoides* Engl. & Diels, *C. psidioides* Welv. and *C. zeyheri* Sond. are used for the treatment of infections and tuberculosis related symptoms in African traditional medicine. In order to verify these uses, extracts were screened for their growth inhibitory effects against *M. smegmatis* ATCC 14468. Ultra-high pressure liquid chromatography coupled to quadrupole time-of-flight mass spectrometry (UHPLC/QTOF-MS) and GC-MS were used to investigate the polyphenolic composition in the active extracts. The lowest minimum inhibitory concentration (MIC), 625 µg/mL, was shown by a methanol extract of the stem bark of *C. psidioides*. A butanol extract of *C. psidioides* gave large inhibition zone diameters (IZD 21 mm) and inhibited 84% of the mycobacterial growth at 312 µg/mL. Combretastatin B-2 and dihydrostilbene derivatives were present in the methanol extract of *C. psidioides*, whereas the butanol extract of this species contained punicalagin, corilagin, and sanguiin H-4. Methanol and butanol extracts of the stem bark of *C. padoides* gave large inhibition zone diameters (IZD 26.5 mm) and MIC values of 1250 and 2500 µg/mL, respectively. *C. padoides* contained an ellagitannin with a mass identical to punicalagin ([M-H]^−^ 1083.0587) and a corilagin like derivative ([M-H]^−^ 633.0750) as well as ellagic acid arabinoside and methyl ellagic acid xyloside. A butanol extract of the roots of *C. zeyheri* showed mild antimycobacterial activity and contained a gallotannin at *m*/*z* [M-H]^−^ 647.0894 as the main compound along with punicalagin and three unknown ellagitannins at *m*/*z* [M-H]^−^ 763.0788, 765.0566, and 817.4212. Our results indicate that the studied species of *Combretum* contain phenolic and polyphenolic compounds with possible potential as leads for antimycobacterial drugs or as adjuvants for conventional anti-TB drugs.

## 1. Introduction

Tuberculosis (TB) continues to be of global concern [1]. Worldwide, TB is among the top ten causes of death, and according to World Health Organization [1], 1.2 million people died from TB in 2018. Developing countries have significantly higher incidents of TB due to malnutrition, poverty, and crowded settings [2]. In the African region, the incidence rate of TB is estimated to be 363 per 100,000 compared to 4.4 per 100,000 in the United States [3,4]. Moreover, up to 22.4% of the population in Africa is infected with latent TB [5]. In Sub-Saharan Africa, the HIV/AIDS epidemic has contributed to create a large reservoir of TB susceptible individuals [6,7,8,9].

Drug-resistant TB continues to be a global health threat and multidrug-resistant (MDR-TB) and extensively drug-resistant (XDR-TB) strains of *M. tuberculosis* are common in Africa [1,10]. Interruption of TB medication due to long treatment courses of 6–9 months has been thought to be the primary contributor to the emergence of drug resistant TB [2,11,12]. The multidrug-resistant strains of *M. tuberculosis*, which are resistant to the two first-line TB drugs, rifampicin and isoniazid, are estimated to result in up to 91% mortality among AIDS patients [13,14]. MDR-TB therapy is more costly, prolonged, and toxic than conventional TB therapy since several drugs, often eight to nine, are used in combination and, in addition, the cure rates are uncertain [15]. The need for finding new and more efficient low-toxic anti-tuberculosis scaffolds is therefore urgent [15,16].

Efforts to identify new anti-TB drugs have been made by screening pharmaceutical library collections, but the efficiency has been poor due to limited chemical diversity in these collections [17]. There is a re-emergence in the interest of natural products as providers of novel structures for antibacterial leads [18]. Natural products, and among them plant derived products, are rich in chemical diversity and thus new anti-TB compounds might be found from plants, and especially from plants used in traditional medicinal systems for TB related symptoms, such as chronic cough, bloody cough, and chest pain [19,20]. Plant derived compounds might act on other sites with other mechanisms of actions than the conventional antibiotics [21]. A number of antimycobacterial compounds have been explored from plants, among them (+)-calanolide A and abruquinone B with MIC 3.13 and 12.5 µg/mL, respectively, against *M. tuberculosis* [18,22,23].

Tropical rain forests, savanna and Miombo woodland forests and other forest biomes in Africa have a high biodiversity, which increases the possibilities for finding new scaffolds for antimicrobial drugs [24,25]. Moreover, there is still a large number of plant species, endemic to Africa, which have not been investigated for their antimicrobial potential [21]. The genus *Combretum* (Combretaceae) comprises about 250–370 species of trees, shrubs, and woody climbers in tropical and subtropical regions of Africa, the Arabic peninsula, Madagascar, India, Asia, Australia, and America [26,27]. The genus has its greatest species diversity in Africa with roughly 300 species [28], and an estimated 163 species occur in tropical Sub-Saharan Africa [29,30]. *Combretum* species occur in a variety of habitats, including Miombo woodlands, dry woodlands, riverine forests, and savannas [31]. Species of *Combretum* have characteristic 4–5 winged fruits, which are important for species characterization [31]. Many species resemble each other closely to their morphology and are therefore usually perceived as a common “*Combretum*-group” with the vernacular name “Mlama”, by traditional healers in Tanzania, although separate vernacular names are sometimes used to distinguish between species [32]. At least twenty-four species of *Combretum* have documented uses in African traditional medicine [33]. Species of *Combretum* are commonly used as decoctions, infusions, and macerations for the treatment of infectious diseases and their symptoms such as diarrhea, cough, fever, chest-pain, and vomiting [33,34,35,36,37,38,39,40,41]. Some species of *Combretum* are reported to be used specifically for tuberculosis, such as *C. hereroense*, *C. molle* and *C. collinum* [2,33,41,42]. The frequent use of *Combretum* spp. in traditional medicine for the treatment of infectious diseases indicates that they contain valuable (novel) antimicrobial compounds.

De Morais Lima et al. [43] give a comprehensive review on antimicrobial and other biological activities of African and Asian *Combretum* spp. According to this review, there is a wealth of studies concerning the growth inhibitory activities of various species of *Combretum* against a number of gram-positive and gram-negative bacteria. However, fewer investigations have been performed involving mycobacteria. Most of these investigations were performed using extracts and most of them involved other mycobacterial species than *Mycobacterium tuberculosis*.

Several compound classes and among them unusual compounds [44] have been characterized from species of *Combretum*, such as tetra- and pentacyclic triterpenes and their desmosides [45,46,47,48,49,50,51,52,53,54], cyclobutane chalcone dimers [55], cycloartane triterpenes [56], diarylpropanes [57,58], flavonoids [59,60], substituted bibenzyls and 9,10-dihydrophenanthrenes [61,62,63,64,65,66,67,68], alkaloids and flavonoid alkaloids [69,70], ellagic acid derivatives [71,72,73], ellagitannins [13,60,74,75,76], and gallotannins [60].

Some recent references on phytochemicals from *Combretum* spp. indicate that the genus contains compounds with topoisomerase activity, such as myricitrin, a flavonoid rhamnoside that was isolated from *Combretum lanceolatum*, a Brazilian medicinal plant [77]. Topoisomerase I is essential for the viability of *M. tuberculosis* and is therefore considered an important drug target for antimycobacterial compounds.

To date, the most active antimycobacterial compound in the genus *Combretum*, the diarylpropane, 1-(2-hydroxy-4-methoxyphenyl)-3-(4-hydroxy-3-methoxyphenyl)propane, was isolated from the Asian species *Combretum griffithii* and inhibited the growth of *M. tuberculosis* with a MIC value of 3.13 µg/mL [57,58]. Moreover, the ellagitannin punicalagin, isolated from an acetone extract of the stem bark of *Combretum molle* gave mild growth inhibitory effects against strains of *M. tuberculosis* [13]. Cholest-5-en-3-ol, 2-phyten-1-ol, gallocatechin, and apigenin isolated from *Combretum paniculatum* were active against *Mycobacterium vaccae* [73].

The aim of the present study was to investigate the antimycobacterial effects of the crude methanolic extracts of *Combretum psidioides*, *C. fragrans,* and *C. zeyheri* and their liquid partition fractions on the fast-growing model bacterium for tuberculosis, *Mycobacterium smegmatis*. The species of *Combretum* were selected based on their frequent uses in African traditional medicine to treat bacterial infections and symptoms related to them, such as cough (Table 1), as well as due to the scarcity or absence of antimycobacterial studies on these particular African species of *Combretum*. Moreover, the *Combretum* species chosen have given promising antibacterial and antifungal effects in our previous studies [78,79]. HPLC-DAD fingerprinting of the active extracts was performed, with special emphasis on polyphenols. Ellagitannins were found to occur in a high variety in the studied plants and this compound class was therefore studied in depth. There are a limited number of studies on ellagitannins in *Combretum* species, despite their predominance in decoctions, macerations, and other common traditional medicinal preparations of these plants. Therefore, ellagitannins (and especially their metabolites, the urolithins) are suggested to be important active ingredients in these preparations. We have used HPLC-DAD and UHPLC/QTOF-MS to elucidate UV absorption maxima, retention times and the molecular masses of ellagitannins and other phenolic compounds. In addition, some dihydrostilbenoid compounds of *C. psidioides* stem bark were characterized using GC-MS.

## 2. Results

### 2.1. Antimycobacterial Effects of Extracts and Fractions

Altogether, 26 extracts and solvent partition fractions from *Combretum psidioides*, *C. padoides* and *C. zeyheri*, used for the treatment of bacterial infections and even cough (*C. zeyheri*) in African traditional medicine (Table 1), were screened for their growth inhibitory effects against *Mycobacterium smegmatis* ATCC 14468. *M. smegmatis* has been found to be rather resistant to rifampicin [83]. Therefore, inhibitory effects against this bacterial strain could indicate that the extracts and fractions also to a certain extent might inhibit rifampicin resistant strains of *Mycobacterium tuberculosis*. Moreover, *M. smegmatis* has some virulence genes in common with *M. tuberculosis*, and thus serves as a good model bacterium [84]. The results regarding the antimycobacterial activity of crude methanol extracts and their solvent partition fractions of the investigated species of *Combretum* spp. are shown in Table 2 and Table 3.

*Combretum psidioides* gave the best growth inhibitory effects against *M. smegmatis* of the three species of *Combretum* used in this investigation. The lowest MIC of 625 µg/mL was shown by a methanol extract of the stem bark of this species. This extract also gave a large inhibition zone diameter of 29.0 mm (Table 2 and Table 3) as well as a good total antimycobacterial activity of 313.44 mL/g (Table 3). The total antimycobacterial activity is dependent on the extraction yield and the MIC of a plant extract, and is calculated as the ratio between the yield (in mg/g) and the MIC (in mg/mL) [84]. Moreover, we also found that chloroform and butanol extracts of the stem bark of *C. psidioides* were growth inhibitory against *M. smegmatis*, both giving MIC values of 2500 µg/mL and inhibition zone diameters of 25.5 and 21.5 mm, respectively (Table 2 and Table 3). In addition, although the MIC of the butanol fraction of *C. psidioides* was 2500 µg/mL, this extract was the most effective of the investigated extracts and fractions to inhibit the growth of *M. smegmatis* at lower concentrations and at 312 µg/mL, this extract resulted in an 84% growth inhibition.

**Figure 1:**
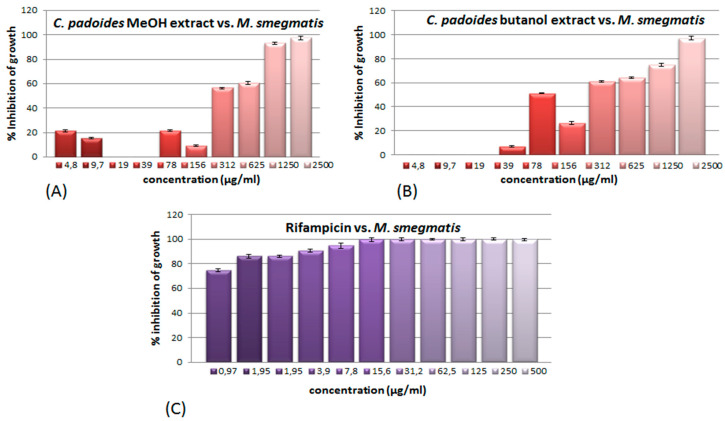
Dose-response bars as percentage inhibition of growth of (**A**) the crude methanol extract and (**B**) the butanol extract of *C. padoides*, and (**C**) rifampicin. Tests were performed in triplicates ±SD.

Antimycobacterial effects were observed for methanol and butanol extracts of the stem bark of *C. padoides*, and both showed inhibition zone diameters of 26.5 mm and an activity index of 0.50 in comparison to rifampicin (IZD 52.5 mm) (Table 2). The MIC value of the crude methanol extract was 1250 µg/mL and for the butanol extract 2500 µg/mL (Table 3) and the growth inhibition of both extracts was dose-dependent for concentrations ranging down to 312 µg/mL (Figure 1). For both the crude methanol and butanol extracts, the growth inhibition was stronger at 78 µg/mL when compared to 156 µg/mL (Figure 1). Although the MIC of the butanol extract was higher than the MIC for the methanol extract, the butanol extract gave stronger growth inhibition than the methanol extract at concentrations below 625 µg/mL, and at 78 µg/mL still 51% of the growth was inhibited (Figure 1).

Compared to *C. padoides* and *C. psidioides, C. zeyheri* gave only mild antimycobacterial effects and a root butanol extract gave the best effect (IZD 23.0 mm) (Table 2).

In comparison to the plant extracts, rifampicin gave an inhibition zone diameter of 52.5 mm and a MIC of 3.9 µg/mL (Table 2, Table 3, Figure 1).

### 2.2. Phytochemistry

#### 2.2.1. Combretum Psidioides

**Figure 2:**
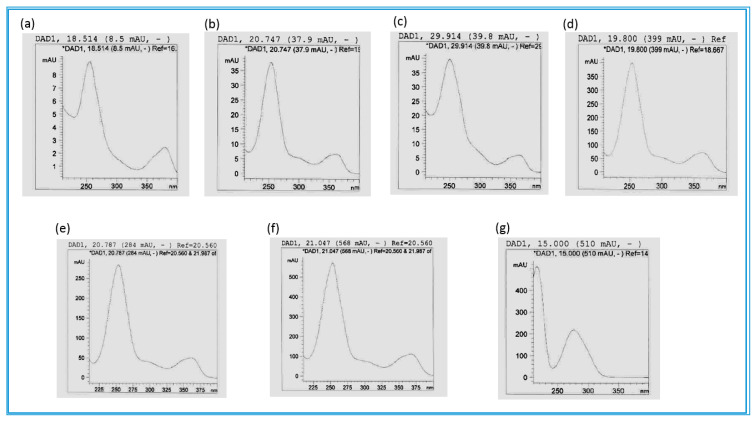
UVλ absorption spectra of ellagic acid derivatives (**a**–**f**) and a gallotannin (**g**) in the investigated species of *Combretum*. (**a**–**c**) *C. zeyheri* at Rt 18.514, 20.747 and 29.914 min; (**d**) *C. psidioides* at Rt 19.800 min; (**e**–**f**), *C. padoides* at Rt 20.707 and 21.047 min; (**g**) the main compound in *C. zeyheri*, a gallotannin at Rt 15.000 min.

**Figure 3:**
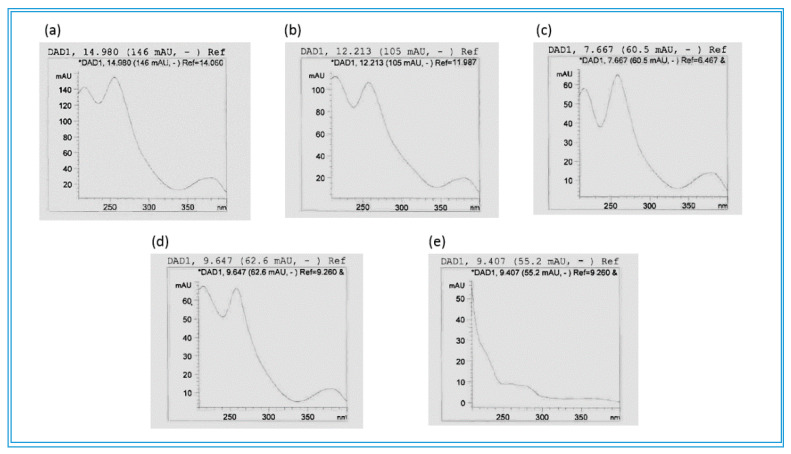
UVλ absorption maxima of (**a**) punicalagin, (**b**) corilagin and (**c**,**d**) two unknown ellagitannins as well as (**e**) epigallocatechin gallate in a butanol extract of *Combretum psidioides* stem bark.

Based on the growth inhibitory results of both the methanol and the butanol extracts of the stem bark of *C. psidioides*, a butanol extract was chosen for phytochemical analysis on its polyphenolic composition using HPLC-DAD and UHPLC/QTOF-MS. Although showing a higher MIC than the methanol extract, the butanol extract was chosen for this analysis since the methanol extract contained a high concentration of condensed tannins that could interfere with the analysis (Figure S1). However, in terms of the ellagitannins and the ellagic acid derivatives composition, the butanol and methanol extracts contained the same compounds (Supplementary Figure S1). The results of the HPLC-DAD and UHPLC/QTOF-MS analysis of the butanol extract of the stem bark of *C. psidioides* are shown in Table 4. 3′-*O*-methyl-4-*O*-(β-d-xylopyranosyl)ellagic acid ( [M-H]^−^ at *m*/*z* 447.0574) was present at Rt 19.85 min (Table 4, Figure 2d). In addition, three other ellagic acid derivatives were identified at Rt 17.56, 18.93, and 24.92 min, showing two clear UVλ absorbance maxima, the first at 254 and the other at 362–380 nm, which is typical for this class of compounds [85]. UHPLC/QTOF-MS data revealed the molecular masses of three tentatively identified ellagitannins; corilagin at Rt 12.24 min ([M-H]^−^ at *m*/*z* 633.0750), punicalagin at Rt 14.96 min ([M-H]^-^ at *m*/*z* 1083.5410, ) and sanguiin H-4 at Rt 8.21 min ([M-H]^−^ at *m*/*z* 633.0746) (Table 4, Figure 3a,b). The thirteen unknown ellagitannins could be identified as ellagitannins based on their UVλ absorption spectra, demonstrating three absorption maxima, characteristic for ellagitannins (Table 4, Figure 3c,d). Epigallocatechin gallate (MW 458.0843) at Rt 9.43 min was tentatively identified according to computer compound libraries available and characteristics such as UVλ absorption maxima data as well as HPLC retention time (Table 4, Figure 3e). To the best of our knowledge, this tea polyphenol has not been reported before in the genus *Combretum*.

**Figure 4:**
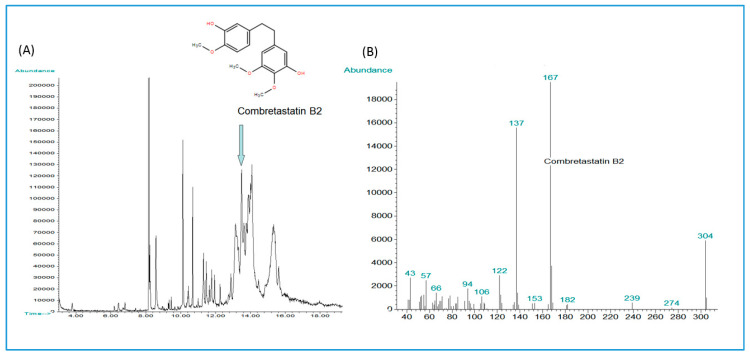
(**A**) Combretastatin B-2 characterized from a methanol extract of the stem bark of *Combretum psidioides* using GC-MS. (**A**) Combretastatin B-2 in a GC-chromatogram and (**B**) mass spectrum of combretastatin B-2 with *m*/*z* 304 as base ion.

Stilbenoid compounds are known to be present in *Combretum* spp. [61,62,63,64,65,66,67,68]. Stilbenes in the methanol extract of the stem bark of *C. psidioides* could contribute to the antimycobacterial effects of this extract (MIC 625 µg/mL). Therefore, a GC-MS analysis was made to study the stilbenoid composition of this extract. This analysis resulted in the characterization of combretastatin B-2 (exact calculated mass for C_17_H_20_O_5_, MW 304.13107) and its dihydrostilbene derivatives (Figure 4, Table 4). To the best of our knowledge, combretastatin B-2 has not been described before in *C. psidioides*, although a number of other bibenzyls were characterized from the heartwood of this plant by Letcher & Nhamo [63].

#### 2.2.2. Combretum Padoides

**Figure 5:**
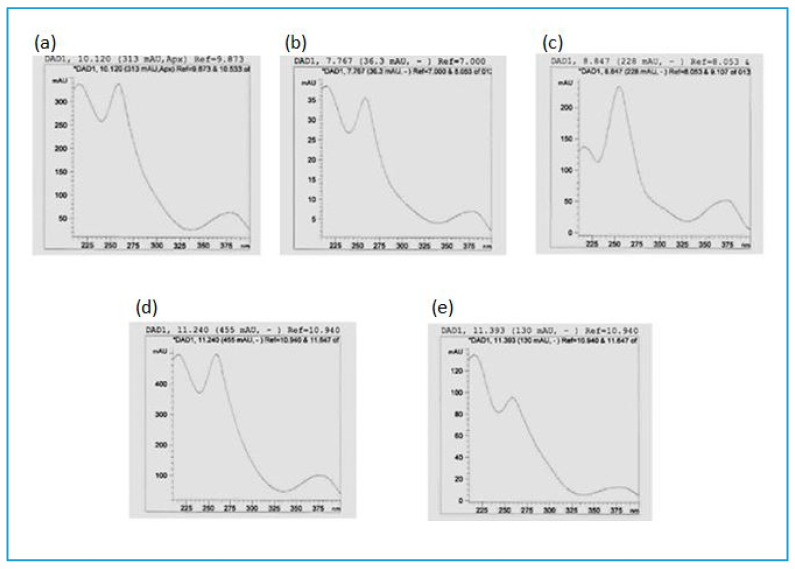
UVλ absorption spectra of ellagitannins in a butanol stem bark extract of *Combretum padoides*. (**a**) A corilagin like ET; (**b**,**c**) unknown ellagitannins; (**d**) punicalagin like ET (either α-punicalagin or punicacortein D); (**e**) an unknown ellagitannin.

Since the HPLC-DAD chromatograms of the methanol and butanol extracts of the stembark of *C. padoides* were almost identical, but with the butanol part showing a slightly cleaner profile (Figure S2), we chose the butanol part for UHPLC/QTOF-MS analysis. In addition, although the MIC was lower for the methanol extract, the IC_50_ value was lower (<312 µg/mL) for the butanol part, which was also a criterium for choosing this extract for the phytochemical analysis. The results of this analysis are shown in Table 5. Altogether twenty-six ellagitannins were tentatively identified based on their retention times, mass spectrometric data and characteristic UV absorbance spectra (Figure 5). The ellagitannin present in the highest concentration, at Rt 10.90 min, resembled punicalagin, giving a [M-H]^−^ molecular ion at *m*/*z* 1083.0587. The retention time of this ellagitannin (10.90 min) was, however, not in agreement with the retention time that we have found before for β-punicalagin (Rt 14.95–15.64 min). Thus, this ellagitannin could be punicacortein D, with the molecular formula C_48_H_28_O_30_, identical to punicalagin, but with a shorter retention time in HPLC-DAD, compared to punicalagin [86]. Punicacortein D has been found in only one other species of the family Combretaceae, *Combretum aculeatum* [86]. However, there is also a possibility that this ellagitannin at Rt 10.90 min would be the α-anomer of punicalagin, which is otherwise identical to β-punicalagin to its mass spectrometric data, but has a shorter HPLC-retention time. Moreover, the α- and β- anomers of punicalagin differ by slight shifts in their UVλ absorbance maxima, due to differences in the position of the OH-group and the H-atom linked to the anomeric C-1 atom [86,87]. However, in our analysis, it was impossible to distinguish α-punicalagin from punicacortein D.

We also found that the stem bark of *C. padoides* contains high concentrations of another ellagitannin at Rt HPLC-DAD 10.11 min, showing a [M-H]^−^ molecular ion at *m*/*z* 633.0750, identical to that of corilagin (Table 5), [88]. The retention time of this ellagitannin however was different from that of corilagin, which we have found at Rt 12.52 min in *Terminalia laxiflora* roots [89] as well as in this investigation in *Combretum psidioides* stem bark at Rt HPLC-DAD 12.24 min (Table 4). Thus, this ellagitannin is tentatively suggested to be an isomer of corilagin. In addition to the mentioned ellagitannins, we identified the masses of four unknown ellagitannins; an ellagitannin at Rt 7.81 min gave a [M-H]^−^ molecular ion at *m*/*z* 466.0264, a second one at tR 8.83 min gave a [M-H]^−^ molecular ion at *m*/*z* 1083.0591, a third one at Rt 11.38 min gave a [M-H]^−^ molecular ion at *m*/*z* 1083.0581 (co-eluting with punicacortein D or α-punicalagin at Rt 10.90 min) and a fourth ET at Rt 20.25 min showing a [M-H]^−^ molecular ion at *m*/*z* 1085.0719 (Table 5). When conducting a literature search for possible ellagitannins with molecular masses of 1086 occurring in *Combretum* spp., we did not find any matches. Instead, we found a reference of an ellagitannin giving a [M-H]^−^ molecular ion at *m*/*z* 1085, found in strawberry fruits [90]. The molecular structure of this ellagitannin was not elucidated, however.

We found five ellagic acid derivatives, at Rt 18.55, 19.96, 20.24, 21.05, and 26.26 min, in the stem bark of *C. padoides*, showing two peaks of UV absorbance, one at 254 and the other at 362–380 nm, typical for ellagic acid based molecules (Table 5, Figure 2e,f). To the best of our knowledge, ellagic acid arabinoside (Rt 20.79 min, [M-H]^−^ at *m*/*z* 433.0391) and methyl ellagic acid xyloside (Rt 21.05 min, [M-H]^-^ at *m*/*z* 447.0564) have not been reported before in *C. padoides.*

Moreover, based on their UV absorption maxima, four gallotannins were identified in the stem bark of *C. padoides*, as well as their precursor, 1-*O*-galloyl-*β*-d-glucose (*β*-glucogallin, Rt 1.58 min) giving a [M-H]^−^ molecular ion at *m*/*z* 331.0685) (Table 5). Glucogallin is known from *Quercus* spp. [91] and from *Emblica officinalis* [92], but to the best of our knowledge not from *Combretum* spp.

#### 2.2.3. Combretum Zeyheri

Since the root part of *C. zeyheri* gave slightly better growth inhibitory effects than the stem bark, this part was chosen for a phytochemical analysis on its polyphenols. Moreover, *C. zeyheri* has not been analysed before for its polyphenolic composition. Our UHPLC/QTOF-MS analysis of the butanol fraction of the roots demonstrated that a gallotannin at Rt 15.008 min, showing a [M-H]^−^ molecular ion at *m*/*z* 765.0566 was the main compound in this extract, giving a peak area of 20.08% (at UV 280 nm, Table 6, Figure 2g). *C. zeyheri* contained more gallotannins and condensed tannins than *C. psidioides* and *C. padoides*. Altogether eight gallotannins and three procyanidins were found based on the UVλ absorption maxima spectra of these compounds. UVλ absorption maxima peaks with two shoulders, one at 216 nm and another at 258–278 nm, could be seen for the gallotannins (Figure 2g), whereas the condensed tannins gave two absorption maxima at 212 and at 272–276 nm (Figure 3e). Moreover, fourteen ellagitannins were identified based on their characteristic UVλ absorption maxima spectra as well as retention times. Of these ellagitannins, punicalagin at Rt 15.64 min ([M-H]^−^ at *m*/*z* 1083.0574) was present in the highest concentrations. In addition, the masses of three unknown ellagitannins were determined; an ellagitannin at Rt 34.99 min ([M-H]^−^ at *m*/*z* 763.0788), another one containing both ellagic and gallic acid units (ellagigallotannin) at Rt 36.93 min ([M-H]^−^ at *m*/*z* 765.0566) and a third at Rt 39.96 min ([M-H]^−^ at *m*/*z* 817.4212). Moreover, five ellagic acid derivatives were present, among which methyl ellagic acid (Rt 29.92 min, ([M-H]^−^ at *m*/*z* 447.0566), dimethyl-ellagic acid-xyloside (Rt 34.24 min, [M-H]^−^ at *m*/*z* 461.0741), and 3,3′-Di-*O*-methyl-4-*O*-(n′′-*O*-galloyl-β-d-xylopyranosyl) ellagic acid (Rt 36.26 min, [M-H]^−^ at *m*/*z* 613.0849) were tentatively identified.

## 3. Discussion

### 3.1. Antimycobacterial Effects of the Extracts of the Studied Species of Combretum in Relation to Other Studies on the Antimycobacterial Effects of Combretum spp

A number of studies demonstrate that extracts of *Combretum* species possess antimycobacterial effects. For example, *Combretum comosum* was found to give growth inhibitory effects against *M. phlei* [93]; *C. brassii* gave antimycobacterial effects against *M. tuberculosis* (MIC 1250 µg/mL) [94]; extracts from *C. platypetalum* and *C. imberbe* gave MIC values of 63–500 µg/mL against *M. smegmatis* and *M. aurum* [95]; acetone extracts of the leaves of *C. hereroense* gave a MIC value of 470 µg/mL against *M. smegmatis* [96]; acetone extracts of the leaves of *C. schumannii* gave a MIC value of 313 µg/mL against *M. madagascariense* and stem bark dichloromethane and root ethanol extracts were effective against *M. indicuspranii* [28]; *C. hartmannianum* leaf ethanol extracts showed strong growth inhibition against *M. aurum* A+ with a MIC value of 190 µg/mL [97] and ethanol extracts of the leaves, bark and root of *C. kraussii* gave MIC values of 195 µg/mL against *M. aurum* A+ [98,99].

In our investigation, methanol and butanol extracts of *Combretum psidioides*, *C. padoides,* and *C. zeyheri* were found to give MIC values from 625 to 2500 µg/mL against *M. smegmatis*. To the best of our knowledge, *C. padoides* and *C. psidioides* have not been studied before for their growth inhibitory effects against *M. smegmatis*. In addition, the roots of *C. zeyheri* have not been explored before for their antimycobacterial effects.

Contrary to our result, Luo et al. [100] reported that stem bark extracts of *C. zeyheri* are not antimycobacterial against *M. smegmatis* and *M. tuberculosis*. However, Luo et al. [100] defined all extracts as not active with MIC values exceeding 125 µg/mL and the MIC results for *C. zeyheri* were not shown. Moreover, Magwenzi et al. [95] found that a leaf extract of *C. zeyheri* was not active against *Mycobacterium smegmatis*. This could be due to differences in growth inhibitory activities between different organs in *C. zeyheri*, so that the stem bark and roots might contain more and perhaps different active compounds than the leaves, since we have seen that especially butanol and methanol extracts of the stem bark and roots of *C. zeyheri* give growth inhibitory effects against *M. smegmatis*. However, an alkaloid enriched leaf extract of *C. zeyheri* was found to inhibit the growth of *M. smegmatis* with a MIC value of 125 µg/mL and the growth inhibition was concentration- and time dependent [101]. Moreover, the same authors also discovered that this alkaloid extract of *C. zeyheri* was a potent inhibitor of efflux pumps in *M. smegmatis*. Thus, *C. zeyheri* (and other African *Combretum* spp.) might contain a number of alkaloids with antimycobacterial properties yet to be explored.

### 3.2. Ellagitannins in the Species of Combretum and Their Suggested Impact on the Antimycobacterial Effects of These Species

**Figure 6:**
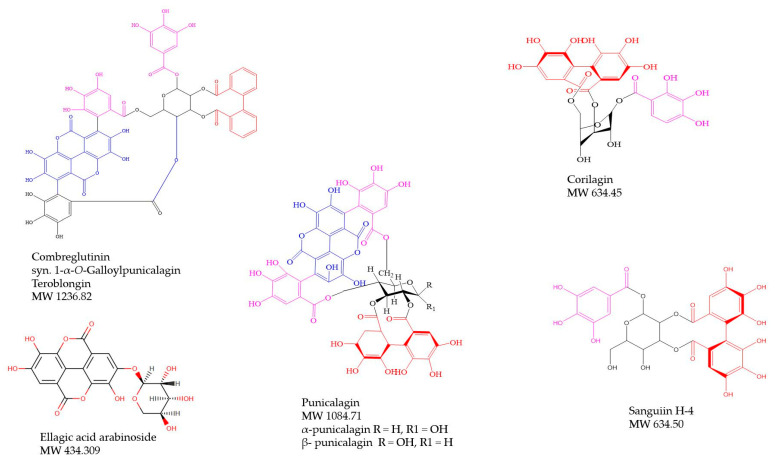
Ellagitannins and ellagic acid arabinoside from the studied species of *Combretum*.

Although some *Combretum* spp. have been found to be rich in ellagitannins and their derivatives as well as other hydrolysable tannins [13,74,86] (Figure 6), these compound categories and their possible potential role as antimicrobials have not been studied in detail in this genus. This could partly be due to the poor bioavailability of ellagitannins, although they could have potential as topically administered antimicrobials or in the gut as well as in combinations with conventional antibiotics or via their metabolites, the urolithins. Among the few studies available on ellagitannins in *Combretum* spp. is the study of Jossang et al. [74], who characterized 2,3-(*S*)-hexahydroxydiphenoyl-d-glucose (MW 482.34), punicalin (MW 782.52), punicalagin (MW 1084.71), and combreglutinin (MW 1236.837) from the leaves of the West-African species, *Combretum glutinosum*. Combreglutinin is composed of one extra galloyl group compared to punicalagin (Figure 6).

In our study, especially butanol and methanol extracts of the stem bark of *C. psidioides* and *C. padoides* and the roots of *C. zeyheri*, enriched in ellagitannins and gallotannins and their monomers (ellagic acid and gallic acid derivatives), gave good antimycobacterial effects. Thus, the antimycobacterial effects of these extracts are suggested to be partly due to these compound classes, and for the ellagitannins perhaps most likely via their urolithin metabolites [86]. This is supported by Coulidiati et al. [102] who demonstrated that tannins, and especially hydrolysable tannins (ellagitannins and gallotannins), predominate in n-butanol extracts of *Combretum sericeum* and these compounds were suggested to be responsible for the antimicrobial activity of the extracts.

We found that punicalagin (Figure 6) is present in the stem bark of *Combretum psidioides* and in the roots of *C. zeyheri*. To the best of our knowledge, there are no previous reports on the occurrence of punicalagin in the mentioned *Combretum* species. Previously, apart from *C. glutinosum*, punicalagin was found in *Combretum molle* [13] and both the α- and β-punicalagin anomers were characterized in *Combretum aculeatum* [86]. Punicalagin isolated from the stem bark of *C. molle* gave growth inhibitory effects against *M. tuberculosis* typus humanus ATCC 27,294 and against a clinical strain with MIC values of 600 and 1200 µg/mL, respectively [13] and was the first ellagitannin reported to possess antimycobacterial effects. We therefore suggest that part of the antimycobacterial activities of the butanol extracts of *C. psidioides* stem bark and *C. zeyheri* roots, reported in this paper, might be due to this ellagitannin, and perhaps in combinations with the other ellagitannins in these extracts. However, again, these activities are in vitro, and further studies should be performed on urolithins resulting from the metabolism of punicalagin, and their effects on mycobacterial strains.

Our study revealed the occurrence of corilagin and sanguiin H-4 in *Combretum psidioides* and a corilagin like ellagitannin in *C. padoides* stem bark (Figure 6). To the best of our knowledge, corilagin and/or its isomers and sanguiin H-4 have not been found in *Combretum* species earlier. Corilagin has been reported to occur in various species of the closely related genera *Terminalia* [86,103,104] and *Lumnitzera* (Combretaceae) [105]. Corilagin was found to give good growth inhibitory effects against *S. aureus* with a MIC value of 25 µg/mL [106], and to inhibit the growth of methicillin resistant *S. aureus* [107,108]. Moreover, corilagin increased membrane permeability in *E. coli* and *C. albicans* [109]. In addition, corilagin gave bactericidal effects in vitro against *Klebsiella pneumoniae* [110] and inhibited the growth of *Acinetobacter baumanii* [111]. Interestingly, corilagin was found to potentiate the activity of β-lactam antibiotics 100–2000-fold against methicillin-resistant *S. aureus* via inhibition of the activity of penicillin binding protein 2 [108,112]. However, in our screenings on the effects of pure corilagin on *M. smegmatis* growth, we found that this ellagitannin showed only weak growth inhibition (MIC 1000 µg/mL, Table 3). This could imply that the ellagitannins in the *Combretum*-extracts work together with each other and with other secondary defense compounds to produce antimycobacterial, and perhaps synergistic or additive effects in combinations. Further investigations on the effects of ellagitannins in the *Combretum* species studied in this investigation and their urolithin metabolites, both alone and in combinations with isoniazid and rifampicin, on the growth of *M. smegmatis* and *M. tuberculosis* are warranted.

Since traditional medicines with *Combretum* spp. as ingredients are often prepared as decoctions or macerations, this means that polar ellagitannins and other polyphenols, extracted with hot or cold water are important components in them. Macerations of the stem bark of *C. psidioides*, enriched with polyphenols, are specifically used for the treatment of diarrhea [34] and decoctions of the roots, leaves and stem bark of *C. zeyheri* are used for cough that could be related to TB [35,37,38], which implies that these species and preparations contain antibacterial (and antimycobacterial) compounds. Interestingly, we have seen that the chromatographic profiles of butanol and methanol extracts of the studied *Combretum* spp. resembled those of the corresponding water extracts, with ellagitannins as the main compounds also in water extracts (Figure S3). This could imply that decoctions and macerations of *C. psidioides*, *C. padoides,* and *C. zeyheri*, used in African traditional medicine, would contain ellagitannins with antibacterial (and antimycobacterial) effects (via their urolithins).

### 3.3. Suggested Antimycobacterial Impact of Ellagic Acid Derivatives in the Species of Combretum Used in This Study

According to the results of the present study, all the investigated species of *Combretum* are rich sources of ellagic acid derivatives. We found that tentatively identified 3′-*O*-methyl-4-*O*-(*β*-d-xylopyranosyl) ellagic acid was present in large quantities in a butanol extract of *C. psidioides* stem bark and ellagic acid arabinoside was present in the stem bark of *C. padoides* (Table 4 and Table 5). Moreover, dimethyl-ellagic acid xyloside and 3,3′-di-*O*-methyl-4-*O*-(n′′-*O*-galloyl-β-d-xylopyranosyl) ellagic acid were tentatively characterized in a butanol extract of the stem bark of *C. zeyheri* (Table 6). To the best of our knowledge, these ellagic acid derivatives have not previously been found in the mentioned species of *Combretum*. These ellagic acid derivatives are suggested to contribute to the good antimycobacterial effects presented in this study for *Combretum psidioides, C. padoides* and *C. zeyheri* since there are a number of reports on good antimycobacterial effects of ellagic acid derivatives. For example, Kuete et al. [25] reported that 3,4′-di-*O*-methylellagic-acid-3′-*O*-β-d-xylopyranoside and 4′-*O*-galloyl-3,3′-di-*O*-methylellagic acid 4-*O*-β-d-xylopyranoside, isolated from the stem bark of *Terminalia superba* gave promising growth inhibitory effects against *Mycobacterium smegmatis* and *M. tuberculosis* H37Rv as well as a clinical strain of *M. tuberculosis*, showing MIC values between 4.88 and 9.76 µg/mL. Moreover, ellagic acid derivatives, such as 3,3′-di-*O*-methyl-ellagic acid, has been found to inhibit the synthesis of mycolic acid, an important cell wall constituent in *Mycobacterium* spp. [113] and pteleoellagic acid and some other ellagic acid derivatives revealed good in silico effects on their docking capacity to enzymes important for the biogenesis of the mycobacterial cell wall [114]. Digalloyl-rhamnopyranosyl ellagic acid and diellagic lactone isolated from the leaves of *Terminalia brownii* did not show growth inhibitory effects against *Mycobacterium intracellulare*, but showed good growth inhibition against other bacteria such as *Pseudomonas aeruginosa* (IC50 8.8. and 8.4 µg/mL, respectively) [115]. In summary, the above-mentioned findings warrant further studies on the antimycobacterial effects of ellagic acid derivatives in African species of *Combretum*.

### 3.4. Stilbenes in Combretum Psidioides and Their Possible Antimycobacterial Effects

The present study demonstrated that the bibenzyl, combretastatin B-2, and some related dihydrostilbene derivatives occurred in a methanolic stem bark extract of *Combretum psidioides* (Figure 4). Earlier, fourteen phenanthrenes and some bibenzyl derivatives (not including combretastatin B-2) have been characterized from the stem bark of *C. psidioides* [63], but these compounds were not investigated for their antibacterial effects. Combretastatins and phenanthrenes are known to occur in some other species of *Combretum*, the most wellknown source being the stem bark of the South African species *C. caffrum* from which a series of antineoplastic combretastatins, among them combretastatin as well as combretastatins A-1, A-2, A-3, B-1, B-2, B-3, and B-4 have been isolated and characterized [65,116]. Some work has been performed on the antimicrobial effects of stilbenoids and phenanthrenes from *Combretum* species: Combretastatin B-5 from the leaves of *C. woodii* gave good antibacterial effects against *S. aureus* with a MIC of 16 µg/mL [67] and phenanthrenes from *C. collinum*, *C. hereroense* and *C. apiculatum* gave a MIC value of 25 µg/mL against *Mycobacterium fortuitum* [117]. These results warrant further studies on the antibacterial and antimycobacterial effects of stilbenoids and phenanthrenes from the *Combretum* species used in this study as well as from African *Combretum* spp. in general.

### 3.5. Epigallocatechin Gallate

Our results indicated that the green tea polyphenol, epigallocatechin gallate, was present in a crude methanol extract of the stem bark of *C. psidioides* (Table 4). This compoud has not been found before in *C. psidioides*. Interestingly, epigallocatechin gallate has been shown to affect cell wall integrity of *M. smegmatis* mc2155, and is suggested to be a good prophylactic agent for TB [118]. In addition, it was found that epigallocatechin gallate inhibits the survival of *M. tuberculosis* within human macrophages [119]. Moreover, (−)-epigallocatechin gallate has been found to repress the expression of transcription factor lasB, involved in the quorum sensing system of *Pseudomonas aeruginosa* [120]. Our study suggests that more species of *Combretum*, used for TB in African traditional medicine, should be explored for their contents of epigallocatechin gallate. Epigallocatechin gallate could be one of the effective antimycobacterial compounds in the stem bark of *C. psidioides*.

### 3.6. Extraction Yield and Its Impact on the Total Antimycobacterial Activity of the Extracts of the Species of Combretum Used in This Study

When a traditional medicinal plant is evaluated for its usefulness, it is important to measure the total activity of its extract or isolated fractions/compounds [84]. The total activity is a measure of the extraction yield of the extracts/fractions divided to the antimicrobial activity of the extracts/fractions (mg extraction yield/MIC in mg/mL) and indicates the volume in ml to which the active compounds in 1 g of the plant material can be diluted and still inhibits bacterial growth [84].

**Figure 7:**
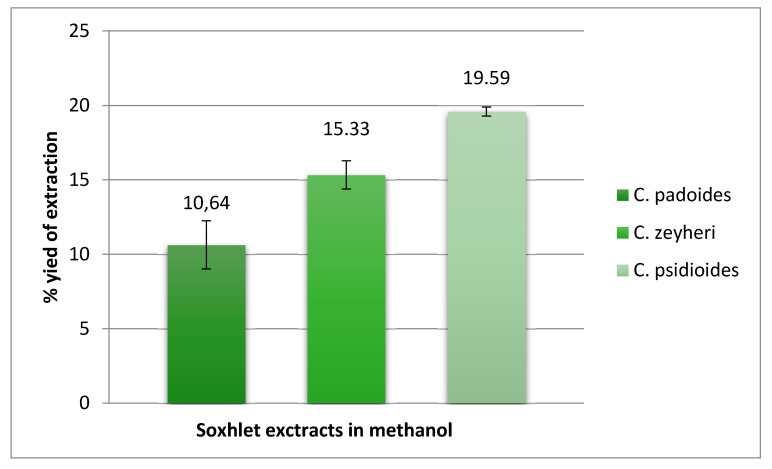
Percentage of extraction yields of crude methanol extracts resulting from Soxhlet extraction of the stem bark of *Combretum padoides* and *C. psidioides* and the roots of *C. zeyheri.* Results are presented as the mean of three extractions ± SD.

We found that methanol extraction of the stem bark of *C. psidioides* results in a high extraction yield of 19.59% (195.9 mg extracted from 1 g plant material) compared to 10.64% and 15.33%, for *C. padoides* and *C. zeyheri*, respectively (Figure 7). Thus, the methanol stem bark extract of *C. psidioides* gives a good total activity of 313.44 mL/g (resulting from dividing the extraction yield to the MIC; 195.9/0.625 = 313.44), and indicates that standardized *C. psidioides* stem bark extracts could have applications as antimycobacterial phytomedicines. The extraction yields for *C. padoides* and *C. zeyheri* obtained in the present study are in agreement with the yields mentioned by Masoko et al. [121]. Moreover, Masoko et al. [121], found that methanol and acetone are suitable solvents for extracting antimicrobials from *Combretum* spp.

**Figure 8:**
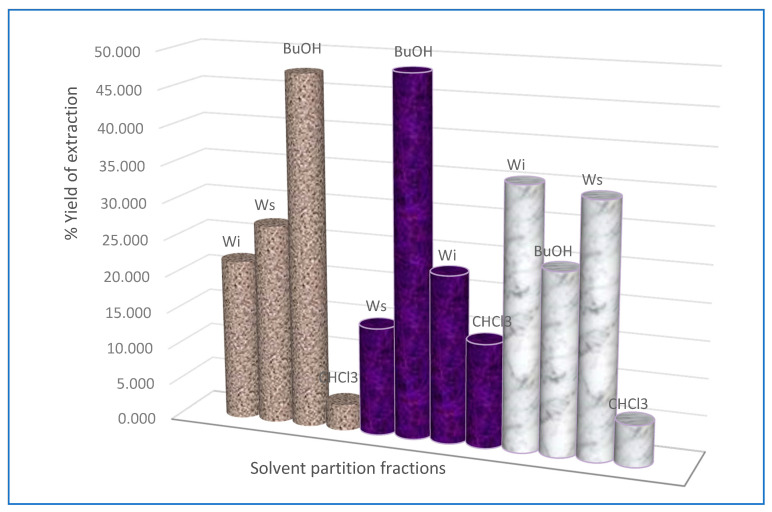
Percentage of exraction yield resulting from solvent partition of crude methanolic extracts of the species of *Combretum* used in this investigation. *C. padoides* stem bark (textured grey bars)*, C. zeyheri* roots (violet texture) and *C. psidioides* stem bark (marble texture). Wi, water insoluble fraction; Ws, aqueous fraction; BuOH, butanol fraction; CHCl3, chloroform fraction.

When the extraction yields of different fractions resulting from solvent partition were compared, it was observed that the butanol fraction gave the highest yield of 47.52% for *Combretum padoides,* and thus resulting in a total antimycobacterial activity of 190.12 mL/g (Table 2, Figure 8). This means that butanol is an optimal solvent for extracting antimycobacterial compounds, such as ellagic acid derivatives and ellagitannins from *C. padoides*. The total extraction yield when using Soxhlet extraction with methanol was, however, observed to be quite low, 10.61% (Figure 7), resulting in a total antimycobacterial activity of 85.12 mL/g of this extract (Table 2).

## 4. Materials and Methods

### 4.1. Plant Material and Ethnopharmacological Background Data

The *Combretum* species were collected from Miombo woodland and riverine forest habitats in Mbeya and Iringa districts in Tanzania in February–March 1999 [32]. Species identification was performed by the botanist Mr. Leonard Mwasumbi, the former superintendent of the Herbarium of the University of Dar-es-Salaam, Tanzania. Voucher specimens are deposited in the Botanical Museum (H) of the Finnish Museum of Natural History in Helsinki, Finland and at the Herbarium of the University of Dar-es-Salaam, Tanzania. The *Combretum* species collected for this study are presented in Table 1 together with data on their ethnopharmacological uses (with special emphasis on cough as a symptom of TB), antimicrobial activity, and their phytochemical composition.

### 4.2. Extraction

#### 4.2.1. Soxhlet Extraction

For each plant sample, 20 g of dried and milled plant material (roots, stem bark, leaves) was extracted with 500 mL MeOH in a Soxhlet apparatus for 4 h. The extracts were reduced to dryness under vacuum using a rotary evaporator, the temperature of the water bath not exceeding +40 °C. For complete drying, the extracts were then further freeze-dried in a lyophilizer for 1–2 days. The resulting residues were redissolved in MeOH (crude extract) to a final concentration of 50 mg/mL for antimycobacterial screening.

#### 4.2.2. Solvent Fractionation

Lyophilized crude Soxhlet methanol extracts (1500 mg) of different plant organs of the *Combretum* species were dissolved in 50 mL distilled water for 30 min using an ultrasonic bath. The extracts were centrifuged in order to separate material, which did not dissolve in water (aqueous insoluble fraction). The aqueous part was used for further fractionation starting with 3 × 25 mL CHCl_3_:EtOH (2:1), whereafter 3 × 25 mL n-BuOH was used. The fractionations thus resulted in CHCl_3_, n-BuOH, aqueous and aqueous-insoluble fractions. The fractions were evaporated to dryness using a rotary evaporator and re-dissolved in MeOH to stock solutions of 50 mg/mL for antimycobacterial testing.

### 4.3. Chromatography and Mass Spectrometry

#### 4.3.1. HPLC-UV/DAD Method I

HPLC analyses for qualitative analysis of extracts of *Combretum* species were performed as described in Fyhrquist et al. [122]. The liquid chromatographic system (Waters 600 E) consisted of a pump and a controller coupled to a 991 PDA detector and a 717 plus automatic sampler under control of Waters Millennium 2000 software. Samples of 20 μL (20 mg/mL inMeOH) were injected. A reversed phase Hypersil BDS-C-18 analytical column (250 mm × 4.6 mm ID 5 μm) was used for the separations. Elution was performed as gradient elution using A) 0.1% formic acid in water and B) 100% acetonitrile. The gradient started with 85% A and ended with 100% B and the length of the runs were 50–60 min. The flow rate was 1 mL/min. UV chromatograms were constructed at 254, 280, and 340 nm. UVλ absorption maxima spectra of the compounds of interest were recorded between 200 and 400 nm using Millennium 2000 software and compared to reference compounds in the computer library.

#### 4.3.2. HPLC-UV/DAD Method II

A second HPLC-method described in Julkunen-Tiitto et al. [123] and Fyhrquist et al., [122], developed especially for the detection of polyphenols, was used. The HPLC-system consisted of a Waters 600 E pump and a controller coupled to a 991 PDA detector and an autosampler under control of Agilent Chemstation software (Waters Corp., Milford, USA). Samples of 10 μL (2 mg/mL in 50% MeOH) were injected. Separations were performed on a reversed phase Hypersil Rp C-18 analytical column (length: 10 mm; ID: 2 mm; particle size 5 µm). Gradient elution was performed by using solvent systems as follows: A) Aqueous 1.5% tetrahydrofuran + 0.25% orthophosphoric acid and B) 100% MeOH. The flow rate was 2 mL/min. UV chromatograms were constructed at 220, 270, 280, 320, and 360 nm. UVλ absorption maxima spectra of compounds, with special emphasis on ellagitannins and ellagic acid derivatives were compared to the Agilent Chemstation library and to literature [88].

#### 4.3.3. UHPLC/Q-TOF MS Method

Ultra-high pressure liquid chromatography coupled to quadrupole time-of-flight mass spectrometry was used as a sensitive method to detect molecular masses with four decimal precision. UHPLC-DAD (Model 1200 Agilent Technologies)-JETSTREAM/QTOFMS (Model 6340 Agilent Technologies) equipped with a 2.1 × 60 mm, 1.7 µm C_18_ column (Agilent technologies) was used for the identification of phenolic compounds based on the method described in Taulavuori et al. [124]. Solvent A was 1.5% tetrahydrofuran and 0.25% acetic acid in HPLC quality water and solvent B was 100% methanol. Gradient runs were as follows: from 0 to 1.5 min, B 0%, from 1.5 to 3 min, 0 to 15% B, from 3 to 6 min, 10 to 30% B, from 6 to 12 min, 30 to 50% B, from 12 to 20 min, 50 to 100% B, and from 20 to 22 min, 100 to 0% B. Negative ion mode were used to acquire the mass spectra depending on the chemical class of the compounds and a mass range from 100 to 2000 *m*/*z* was used. The negative mode was found to be favorable for ellagitannins and ellagic acid derivatives. Pfundstein et al., [88] was used as the main reference for ellagitannins, ellagic acid derivatives and gallotannins.

Mass measurement error (mass accuracy) was calculated according to Brenton and Godfrey [125]: Difference between an individual measurement and the true value ΔMi (in ppm, parts per million) = (M _measured_ − M _calculated_) × 10^6^/M _calculated_, where M _measured_ is the measured mass in QTOF-MS and M _calculated_ is the exact calculated mass according to the molecular formula of the compound. Negative mode of qtof was used, and thus the mass of the hydrogen atom (1.0078) was subtracted from all the calculated masses.

#### 4.3.4. GC-MS Method

The GC-MS analyses were performed on Hewlett-Packard (HP) 5890 GC coupled to an HP quadrupole mass selective detector operated at ionization voltage of 70 eV (EI-mode). The crude methanol extract of the stem bark of *Combretum psidioides* was evaporated and analysed on an NB-54 fused silica capillary column (15 m; 0.20 mm i.d.; Nordion, Finland) using an oven temperature programming from 70 °C to 275 °C at 15 °C/min. A GC-MS library (The Wiley^®^ Registry of Mass Spectral Data, John Wiley & Sons Inc., Electronic division, New York, NY, USA) was used for the elucidation of molecular masses of stilbenoid compounds in the extract.

### 4.4. Assays for Testing Antimycobacterial Activity

#### 4.4.1. Agar Disk Diffusion

An agar disk diffusion method, explained in detail in Fyhrquist et al. [122], was used. *Mycobacterium smegmatis* ATCC 14,468 was grown for five days at +37 °C on Löwenstein-Jensen agar slants (Becton-Dickinson & Company, USA). 200 µL of bacterial culture containing 1.0 × 10^8^ CFU/mL was inoculated on Petri dishes (∅ = 14 cm, Bibby Sterilin, UK) containing 30 mL Middlebrook 7H10 agar (Difco) supplemented with OADC supplement (Difco) as a top layer and 30 mL base agar (Antibiotic agar No 2, Difco) as a bottom layer. Filter paper disks (∅ = 12.7 mm, Schleicher & Schuell) loaded with 200 µL extracts or fractions (50 and 20 mg/mL, respectively), 200 µL rifampicin (10 mg/mL, Sigma-Aldrich) and 200 µL MeOH (as negative control) were allowed to dry completely before placing them equidistantly from each other on the petri dishes. Prior to incubation, the petri dishes were kept in +4 °C for 1 h. The petri dishes were incubated at +37 °C for five days. Each extract/fraction was tested in triplicate and three separate experiments were performed. The diameter of zones of inhibition (IZD) were measured with a caliper and the mean of 3 diameters ± SEM was calculated. Activity index (AI) was measured as the percentage of activity compared to rifampicin, as described by Fyhrquist et al. [122].

#### 4.4.2. Microplate Dilution Method

A modified microplate assay based on Collins & Franzblau [126] and described in Fyhrquist et al. [122] was used for measuring minimum inhibitory concentrations of extracts of *Combretum* spp. *Mycobacterium smegmatis* ATCC 14468 was incubated for five days at +37 °C on Löwenstein-Jensen agar slants and transferred into Dubos broth after completed incubation. The absorbance of the samples was adjusted to 0.1 at 625 nm (approx. 1.0 × 10^8^ CFU/mL) using Dubos broth supplemented with Dubos broth albumin (Difco) and diluted further to 5.0 × 10^5^ CFU/mL. 100 µL of this inoculum was added to the wells of the microplates (Nunc, Nunclone, Denmark). 100 µL of two-fold dilutions of plant extracts (9.76–5000 µg/mL) and of rifampicin (0.9–1000 µg/mL) in Dubos broth or 100 µL Dubos broth (growth control) were added to the wells of the microplate, so that the final volume in the wells was 200 µL, thus containing 2.5 × 10^5^ CFU/mL. In order to measure eventual absorption at 620 nm by the plant extracts or the antibiotic, solvent partition fractions, extracts and rifampicin, alone without bacteria were used as controls and the absorption produced by these samples was subtracted from the corresponding samples containing bacterial cells. The solvent control, MeOH at 5% or less, did not affect the growth of *M. smegmatis*. The microplates were incubated for four days at +37 °C, whereafter turbidity of the wells at 620 nm was measured using a Victor (Wallac, Finland) spectrophotometer. The results were calculated as the mean percentage inhibition of the growth control of three replicate samples ± SEM. The smallest concentration of the extracts and of rifampicin inhibiting 90% or more of the growth of *M. smegmatis* and thus resulting in no visible growth was considered as the MIC.

### 4.5. Calculation of Total Antimycobacterial Activity

The total activity is a measure of the relation of the exraction yield of a plant extract divided to the MIC of that extract. The total activity indicates the volume into which 1 g of plant extract can be diluted without losing its antibacterial activity [84]. The total antimycobacterial activity for plant extracts in this study was calculated as follows:

Total activity (mL/g) = extraction yield of extract A (mg/1000 mg) divided to the MIC of extract A (in mg/mL).

## 5. Conclusions

The results of the present study confirm the antimycobacterial potential of butanol, methanol, and watersoluble extracts of the stem bark of *Combretum padoides* and *C. psidioides* and the stem bark and roots of *C. zeyheri.* The traditional use of decoctions and macerations of *C. zeyheri* for the treatment of cough [81] is justified by these results. The results from this study also suggest that standardized ethanol and water-based extracts of *C. padoides* and *C. psidioides* could be used for the treatment of tuberculosis, although these species are not mentioned to be used for this purpose in African traditional medicine.

The growth inhibitory effects of the butanol, methanol and watersoluble extracts of the *Combretum* species in this study are suggested to be partly due to ellagitannins and ellagic acid derivatives, which are abundantly present in these extracts. However, the therapeutic use of ellagitannins for TB is likely to be limited to their metabolic products, the urolithins, since there is so far only one report on the occurrence of intact ellagitannins in the plasma of rats after a prolonged intake of ET rich foods [127].

The ellagic acid derivatives, and especially the glycosides that we have found in the *Combretum* species studied, could have an interesting potential as new anti-TB drug scaffolds, since some ellagic acid derivatives, such as dimethyl ellagic acid xyloside, that was found in the roots of *C. zeyheri* in this investigation, have been found to possess promising antimycobacterial effects, amongst others as inhibitors of the synthesis of mycolic acid [113].

The results from this study indicate that extracts of *Combretum psidioides*, *C. padoides,* and *C. zeyheri* should be tested against other mycobacteria, and especially *Mycobacterium tuberculosis*, since moderate MIC values against *M. smegmatis* could imply good MIC values against *M. tuberculosis*. The activity guided isolation of active compounds from the butanol extracts, with special emphasis on ellagic acid derivatives, remains to be performed. Moreover, urolithins resulting from the metabolic conversion of the ellagitannins punicalagin, corilagin, and saguiin H-4, should be tested for their antimycobacterial effects.

Lastly, the potential of ellagitannins and ellagic acid derivatives, and other phenolic compounds from the investigated species of *Combretum* spp. should be tested as antibiotic adjuvants to enhance the effects of rifampicin, isoniazid, and other conventional anti-TB drugs.

## Figures and Tables

**Table 1:** Summary of applications in traditional medicine and antimicrobial activity as well as identified compounds of the species of *Combretum* used in this study.

*Combretum* Species	Uses of Plant Species in TM	Way of Preparation in Traditional Medicine	Antibacterial and Antimycobacterial Effects	Identified Compounds
*C. padoides* Engl. & Diels, Thicket bush-willow (P. Fyhrquist & L. Mwasumbi Voucher 1697070)	**Leaves and roots**: For treatment of snakebites, wounds, hookworms, bloody diarrhea, malaria and conjunctivitis [30,37,41].	Water extracts of leaves for snakebites and root decoctions for hookworms. Crushed leaves for wounds [30,37,41].	Extracts of the leaves gave excellent inhibitory effects against *E. coli* and *E. aerogenes* at 0.8 mg/mL [80]; Stem bark extracts gave good antibacterial and antifungal effects [78,79]. 1α,23β-dihydroxy-12-oleanen-29-oic-acid-23β-*O*-α-4-acetylrhamnopyranoside and 1,22-dihydroxy-12-oleanen-30-oic acid from leaves gave antibacterial effects against *S. aureus* and *E. coli* [50]. Crude MeOH extract of stem bark and its BuOH fraction inhibit *Mycobacterium smegmatis* (AI is 50.5% of the inhibitory effect of rifampicin, MIC 1250–2500 µg/mL, IZD 26.5 mm). *	Leaves contain rhamnose based mono- and bi-desmosidic triterpenoids [46,47], leaves contain oleanane-type triterpenoid glycoside, 1α,23β-dihydroxy-12-oleanen-29-oic-acid-23β-*O*-α-4-acetylrhamnopyranoside and two known triterpenoids; 1,22-dihydroxy-12-oleanen-30-oic acid and 24-ethylcholesta-7,22,25-trien-*O*-β-d-glucopyranoside [50]. Punicalagin and a corilagin derivative and twenty-four other unknown ellagitannins, five gallotannins and five ellagic acid derivatives; ellagic acid arabinoside and methyl ellagic acid xyloside in an antimycobacterial BuOH extract of stem bark. *
*C. psidioides* Welw. Velvet bush-willow *C. psidioides*, continued (P. Fyhrquist & L. Mwasumbi Voucher 1697037)	**Leaves and roots**: Aphrodisiac, diarrhea, malaria, back pain, galactogogue [34]. Leaves and roots used for diarrhea, muscle pain, oedema [78].	Decoctions of roots, leaf extracts or leaves mixed with maize porridge (Ugali) for treatment of diarrhea and oedema [78].	Broad-spectrum antibacterial profile in our earlier investigation [78] as well as some antifungal effects [79]. Crude methanol extract and its butanol and CHCl_3_ fractions were growth inhibitory against *Mycobacterium smegmatis* (MIC 625-2500 µg/mL). *	Substituted phenanthrenes and bibenzyls from heartwood and stem bark [63]; Oleanane and ursane pentacyclic triterpene glucosides from root bark [48]. Combretastatin B-2 was tentatively identified in a methanol extract of the stem bark. Sixteen ellagitannins including corilagin and its isomer and punicalagin, six gallotannins including 1,6-di-*O*-galloyl-β-d-glucose, epigallocatechin gallate, four ellagic acid derivatives and protocatechuic acid were identified in a butanol extract of the stem bark. *
*C. zeyheri* Sond., Large-fruited bush-willow (P. Fyhrquist & L. Mwasumbi Voucher 1697020)	**Leaves, roots and stem bark**: Wounds, **cough (TB?)**, malaria, diarrhea, inflammation, scorpion sting, back pain, dysentery, hook worms, tooth ache, eye lotion [35,37,38]. Diarrhea, cancer (stomach tumors) [78].	Smoke of burnt leaves inhaled for **cough (TB?)**, water extracts of dried leaves for colic, crushed leaves for rheumatism and joint pain [35]; hot water decoctions of roots for diarrhea, dysentery and ankylostomiasis [37]; Pounded roots cooked in porridge for hookworms and dysentery, ground roots cooked and applied to wounds, **root decoctions** for stomach-ache, **cough, pneumonia**, vomiting, stomach ulcers and diarrhea, **leaf infusions** for **cough**, stem bark infusion for leprosy [80]. Roots, leaves and stem bark made into decoctions or mixed in maize porridge for diarrhea and stomach tumors [78].	Stem bark and leaf extracts inhibitory against several bacteria [81,82]; Fruit, stem bark and root extracts show good antibacterial potential. Triterpenoids from leaves evaluated for anti-*Candida* effects; terminolic acid was found to be the most active compound. SAR: oleanane and ursane type triterpenoids were the most active ones [54]. We found that extracts of stem bark and roots inhibit the growth of *Mycobacterium smegmatis*, the BuOH fraction of the roots being especially active (IZ 23 mm).*	Triterpenoids and saponins from the leaves [47]; Three unidentified antimicrobial compounds were isolated from the leaves and stem bark [82]; Ursolic acid, maslinic acid, 2α,3β-dihydroxyurs- 12-en-28-oic acid, 6β-hydroxymaslinic acid and terminolic acid from leaves [54]. A root butanol extract contained six ellagic acid derivatives including methyl-ellagic acid xyloside, di-methyl-ellagic acid xyloside and 3,3′-Di-*O*-methyl-4-*O*-(n′′-*O*-galloyl-β-d-xylopyranosyl) ellagic acid, fifteen ellagitannins including punicalagin and nine gallotannins including hexagalloylglucose.*

Our results presented in this paper *; AI, activity index in % activity of rifampicin; MIC, minimum inhibitory concentration; IZD, inhibition zone diameter in mm; TB, tuberculosis; TM, traditional medicine. In brackets the voucher numbers of the collected specimen used in this study. Plant uses for symptoms related to TB marked with bold text.

**Table 2:** Antimycobacterial effects of extracts and their solvent partition fractions of species of *Combretum* collected in Tanzania by the first author in spring 1999. Results obtained with an agar diffusion method. Activity index (AI) in relation to rifampicin.

*Combretum* Species, Extracts and Fractions	*M. Smegmatis ATCC 14468*	AI
*C. padoides ^S^* *^Cr^*	26.5 ± 0.5	0.50
*C. padoides ^WI^*	18.0 ± 2.0	0.34
*C. padoides ^BuOH^*	26.5 ± 0.5	0.50
*C. padoides ^CHCl3^*	17.5 ± 0.5	0.33
*C. zeyheri ^S^* *^Cr 12^*	20.5 ± 0.5	0.39
*C. zeyheri ^WI^*	0.0	0.00
*C. zeyheri ^Ws^*	17.0 ± 3.0	0.32
*C. zeyheri ^BuOH^*	19.0 ± 2.0	0.36
*C. zeyheri ^CHCl3^*	14.0 ± 0.0	0.27
*C. zeyheri ^S^* *^Cr 43^*	21.0 ± 0.0	0.40
*C. zeyheri ^WS^*	18.5 ± 0.5	0.35
*C. zeyheri ^WI^*	17.5 ± 0.5	0.33
*C. zeyheri ^BuOH^*	20.5 ± 0.5	0.39
*C. zeyheri ^CHCl3^*	0.0	0.00
*C. zeyheri ^R^* ^*Cr 33*^	16.0 ± 0.8	0.30
*C. zeyheri ^WS^*	0.0	0.00
*C. zeyheri ^WI^*	16.0 ± 1.6	0.30
*C. zeyheri ^BuOH^*	23.0 ± 1.6	0.44
*C. zeyheri ^CHCl3^*	14.0 ± 0.0	0.27
*C. psidioides ^S^* *^Cr^*	29.00 ± 0.5	0.53
*C. psidioides ^BuOH^*	21.5 ± 2.0	0.41
*C. psidioides ^WI^*	0.0	0.00
*C. psidioides ^CHCl3^*	25.5 ± 0.7	0.48
*C. psidioides ^L Cr^*	0.0	0.00
*Rifampicin*	52.5 ± 0.5	1.00

R, roots; S, stem bark; F, fruits; L, leaves; Cr, crude methanol extract; WS, aqueous fraction; WI, aqueous insoluble fraction; BuOH, butanol fraction; CHCl3, chloroform fraction. 12, 43 and 33 are different individuals of *C. zeyheri*. Two hundred µL extracts/fractions (50 mg/mL) and rifampicin (10 mg/mL) were applied on filter paper discs. Diameter of inhibition zones (IZD) in mm as mean of triplicates (*n* = 3) ± SEM of three experiments. Most promising results indicated by bold text and the best result underlined.

**Table 3:** Minimum inhibitory concentrations and total antimycobacterial activities of crude methanol extracts of *Combretum* spp. and their solvent partition fractions against *Mycobacterium smegmatis*. Pure compounds, such as corilagin and ellagic acid, representative for compounds occurring in the extracts were also tested.

Extracts	*M. Smegmatis* ATCC 14468	Total Activity (mL/g)
***C. psidioides* stem bark:**		
crude methanol extract	625 (IC_94_)	313.44
butanol soluble fraction	2500 (IC_95_)	98.76
chloroform soluble fraction	2500 (IC_90_)	22.89
***C. padoides* stem bark:**		
crude methanol extract	1250 (IC_93_)	85.12
butanol soluble fraction	2500 (IC_97_)	190.12
**Pure compounds:**		
Corilagin *	1000 (IC_94_)	
Ellagic acid **	500 (IC_98_)	
**Rifampicin**	3.90 (IC_91_)	

In brackets the mean percentage of the bacterial growth inhibited by indicated concentration and resulting in no visible growth (IC, inhibitory concentration). * In butanol extract of *C. psidioides*; ** Not present in the extracts as such but as ellagic acid glycosidic derivatives. The total antimycobacterial activity (mL/g) is and indication on the degree to which 1 g of an extract can be diluted without losing its antimycobacterial activity and is calculated as: extraction yield in mg extracted material/mg plant powder divided to the MIC of the extract [83].

**Table 4:** Polyphenolic profile of a butanol extract of the stem bark of *Combretum psidioides*. Data recorded using HPLC-DAD and UHPLC coupled to quadrupole time of flight mass spectrometry. Combretastatin B-2 was identified using GC-MS*.

Compounds	Molecular Formula	Rt HPLC-DAD or GC* (min)	Rt UHPLC (min)	Measured [M-H]^−^ (*m*/*z*) or * GC-MS	Exact Mass (calc.)	ppm Value	UVλmax (in MeOH)	Peak Area % (280 nm)
Gallic acid	C_7_H_6_O_5_	1.636	1.334	169.0149	170.0213	8.2834	216, 272	9.2848
Flavonoid?		1.933					210, 224, 278	0.1800
Protocatechuic acid	C_7_H_6_O_4_	3.718	2.733	153.0196	154.0264	6.5352	210, 218, 260, 294	4.6595
*O*-hydroxycinnamic acid like		4.628					210, 230, 280, 310	0.7723
Unknown ellagitannin		7.187					214, 258, 376	
Unknown ellagitannin		7.731					216, 260, 378	4.0268
Sanguiin H-4	C_27_H_22_O_18_	8.208	4.448	633.0746	634.0798	4.1070	216, 258, 376	5.7862
Gallotannin		8.979					258, 218	0.1717
Epigallocatechin gallate	C_22_H_18_O_11_	9.427			458.0843		210, 276	0.5409
Unknown ellagitannin		9.673					216, 258, 378	2.5987
1,6-Di-*O*-galloyl-β-d-Glucose	C_20_H_20_O_14_	10.022	4.700	483.0801	484.0846	6.8312	216, 276	2.7681
Unknown ellagitannin		10.406					214, 252, 384	0.1968
Unknown ellagitannin		10.778					216, 260, 374	4.4198
Galloylglucose		10.975					216, 276	2.0395
Unknown ellagitannin		11.178					214, 260, 381	0.0556
Unknown ellagitannin		11.454					218, 256, 378	0.8819
Corilagin	C_27_H_22_O_18_	12.244		633.0750	634.0798	4.7388	215, 258, 380	5.7058
Epigallocatechin like (flavan-3-ol)		13.391					210, 282, 320	0.5197
Combretastatin B-2	C_17_H_20_O_5_	*13.489		*304	304.1311	6.9049		
Galloylglucose		14.281					216, 276	4.2740
β-Punicalagin	C_48_H_28_O_30_	14.956	5.427	1083.1541	1084.0654	25.6045	218, 256, 380	6.9297
Epigallocatechin like		17.368					210, 284	
Ellagic acid derivative		17.565					254, 380	3.3214
Gallotannin		17.591					216, 282	
Ellagic acid derivative		18.935					254, 362	0.3382
3′-*O*-methyl-4-*O*-(β-d-xylopyranosyl) ellagic acid (main compound)	C_20_H_16_O_12_	19.846	8.201	447.0574	448.0636	3.5790	254, 364	8.4825
Gallotannin (not PGG)		21.184		939.1000			214, 280	0.1463
Unknown ellagitannin		21.815					218, 248, 368	0.5505
Ellagic acid derivative		24.922					254,362	3.1564
Unknown ellagitannin		26.444					210, 264, 398	0.0953
Unknown ellagitannin		27.163					218, 254, 360	0.3147
Unknown ellagitannin		28.605					218, 256, 362	2.0192
Unknown ellagitannin		31.560					216, 256, 360	1.8907
Unknown ellagitannin		3.688					218, 256, 360	0.9801
Hexagalloylglucose	C_48_H_36_O_30_	38.299			1092.1278		218, 280	0.1879

Rt (min), retention times obtained from HPLC-DAD and UHPLC-DAD; [M-H]^−^, base ion at negative mode. Percentage peak area from HPLC-DAD chromatograms was acquired at 280 nm. PGG, pentagalloylglucose. * results obtained with GC-MS.

**Table 5:** Polyphenolic profile of a butanol extract of the stem bark of *Combretum padoides*. Data recorded using HPLC-DAD and UHPLC coupled to quadrupole time of flight mass spectrometry.

Compounds	Molecular Formula	Rt HPLC-DAD (min)	Rt UHPLC (min)	Measured [M-H]^−^ (*m*/*z*)	Exact Mass (calc.)	ppm Value	UVλmax (in MeOH)	Peak Area % (280 nm)
1-*O*-galloyl-*β*-d-glucose (syn. *β*-glucogallin)	C_13_H_16_O_10_	1.577		331.0685	332.0738	7.5514	216, 276	0.1894
Gallic acid	C_7_H_6_O_5_	1.763	1.306	169.0157	170.0213	13.0167	216, 272	1.2354
Ellagitannin		3.245					215, 260, 380	1.8848
Ellagitannin		7.815	3.170	466.0264			214, 258, 380	3.1453
Ellagitannin		8.408					216, 258, 376	1.4324
Ellagitannin		8.829	3.387	1083.0591			216, 256, 374	8.3917
Gallotannin		9.370					216, 252	2.6083
Ellagitannin		9.740					210, 258, 380	0.7464
Corilagin derivative	C_27_H_22_O_18_	10.114	3.768	633.0750	634.0798	4.7388	216, 258, 379	10.9106
Ellagitannin		10.413					220, 256, 374	0.4238
α-Punicalagin anomer or Punicacortein D	C_48_H_28_O_30_	10.903	3.853	1083.0587	1084.0654	1.0156	216, 260, 376	14.9380
Ellagitannin		11.382	3.903	1083.0581			216, 258, 376	3.9407
Ellagitannin		11.764					216, 258, 380	0.1157
Ellagitannin		12.073					218, 258, 378	4.6576
Ellagitannin		12.385					224, 256, 374	0.7334
Ellagitannin		12.619					210, 260, 382	0.2170
Ellagitannin		12.858					214, 256, 378	2.7210
Unknown		13.373					214, 248, 292	0.1398
Flavonoid; Ampelopsine like		13.643					210, 280	0.5307
Ellagitannin		14.054					218, 258, 368	0.1152
Ellagitannin		14.500					220, 260, 376	0.8036
Flavonoid		15.137					210, 276, 370	0,9277
Ellagitannin		15.693					216, 256, 380	5.3916
Ellagitannin		16.015					210, 256, 364	0.2770
Ellagitannin		16.219					216, 260, 378	0.7760
Ellagitannin		16.849					216, 284, 382	0.3067
Ellagitannin		17.668					218, 260, 378	0.8513
Gallotannin		17.975					218, 280	0.2328
Ellagic acid derivative		18.556					254, 380	0.4644
Stilbene like compound?		18.809					210, 235, 382	0.9999
Gallotannin		18.991					210, 280	0.4259
Ellagic acid derivative		19.960					254, 362	0.2292
Ellagitannin		20.253	4.502	1085.0719			210, 254, 362	0.5726
Ellagic acid arabinoside	C_19_H_14_O_12_	20.792	8.316	433.0391	434.0480	-2.5402	254, 362	1.6928
3′-*O*-methyl-4-*O*-(β-d-xylopyranosyl) ellagic acid	C_20_H_16_O_12_	21.055	8.616	447.0564	448.0636	1.3421	254, 368	4.4746
Gallotannin (not PGG)		22.163					214, 284	0.1343
Ellagic acid derivative		26.258					254, 360	1.5195
Ellagitannin		28.596					216, 254, 362	0.1066
Ellagitannin		30.121					216, 254, 362	1.5374
Ellagitannin		33.134					216, 256, 360	2.2061
Ellagitannin		36.269					218, 256, 362	0.8344

Rt (min), retention times obtained from HPLC-DAD and UHPLC-DAD; [M-H]^−^, base ion at negative mode. Percentage peak area was acquired from HPLC-DAD chromatograms at 280 nm.

**Table 6:** Polyphenolic profile of a butanol extract of the roots of *Combretum zeyheri*. Data recorded using HPLC-DAD and UHPLC coupled to quadrupole time of flight mass spectrometry.

Compounds	Molecular Formula	Rt HPLC-DAD (min)	Rt UHPLC (min)	Measured [M-H]^−^ (*m*/*z*)	Exact Mass (calc.)	ppm Value	UVλmax (in MeOH)	Peak Area % (280 nm)
Gallic acid	C_7_H_6_O_5_	1.760	1.173	169.0140	170.0213	2.9583	216, 272	1.1995
Gallic acid derivative		2.191					216, 274	0.6905
Protocatechuic acid	C_7_H_6_O_4_	3.865	2.593	153.0198	154.0264		218, 220, 260, 294	0.1245
Cinnamic acid like		7.025						0.1205
Gallotannin or gallic acid derivative		8.516					216, 274	3.3768
Corilagin isomer		8.835					216, 254, 374	2.8165
Gallotannin		9.325					216, 258	0.1997
Ellagitannin		10.080					218, 258, 378	0.0607
Gallotannin		10.481					216, 275	3.2150
Ellagitannin		11.190					216, 256, 378	0.2296
Gallotannin		11.486					216, 277	2.2688
Ellagitannin		11.984					220, 256, 378	0.3862
Ellagitannin		12.804					216, 256, 380	2.2389
Eriodictyol like		13.899					210, 279	2.2785
Ellagitannin		14.531					222, 258, 380	0.1015
Gallotannin		15.008	5.352	647.0894			216, 276	20.0817
Punicalagin	C_48_H_28_O_30_	15.645	5.485	1083.0574	1084.0654	−0.1847	216, 256, 381	4.3927
Batatasin like		16.068					210, 272	0.0939
Ellagitannin		16.562					256, 372	0.0601
Salidrosid and arbutin like		17.497					217, 274, 276, 298	0.7553
Ellagic acid derivative		18.559	5.718	435.1310			254, 380	0.4557
Lignan?		19.014					210, 272	2.3806
Lignan?		19.462					210, 274	0.0977
Lignan?		20.178					210, 276	3.1006
Ellagic acid derivative		20.773	5.818	581.1903			254, 362	0.4712
Procyanidin B-3 like		23.022					212, 274	2.8707
Ellagic acid derivative		24.693	8.033	435.1310			250, 366	1.4412
Ellagitannin		26.230					210, 254, 360	1.2651
Lignan?		26.778					210, 274	1.3692
Procyanidin B-3 like		27.670					212, 276	1.0954
Stilbene or lignan?		27.956					212, 264	0.8470
Procyanidin B-3 like		28.803					212, 272	1.2008
Methyl-ellagic acid xyloside	C_20_H_16_O_12_	29.922	8.582	447.0566	448.0636	1.7895	210, 274	1.5613
Ellagitannin		31.578						0.0677
Ellagitannin		32.116					216, 256, 366	0.1269
Ellagitannin		33.103					216, 256, 360	0.7444
Di-methyl-ellagic acid xyloside	C_21_H_18_O_12_	34.243	9.931	461.0741	462.0792	5.8559	251, 366	0.9611
Ellagitannin		34.998	10.192	763.0788			226, 268, 370	0.2394
3,3′-Di-*O*-methyl-4-*O*-(n′′-*O*-galloyl-β-d-xylopyranosyl) ellagic acid	C_28_H_22_O_16_	36.257	12.312	613.0849	614.0900	4.4039	218, 256, 358	0.2287
Gallotannin or ellagitannin according to the UV abs max		36.931	12.829	727.0566; 765.0566			216, 252, 366	1.1001
Hexagalloylglucose	C_48_H_36_O_30_	39.583			1092.1278		216, 278	5.0497
Ellagitannin		39.956	13.994	817.4212			210, 252, 363	0.4127
Gallotannin Ellagitannin?		44.414	17.000	725.4118			216, 274	0.3647

Rt (min), retention times obtained from HPLC-DAD and UHPLC-DAD; [M-H]^−^, base ion at negative mode. Percentage peak area was acquired from HPLC-DAD chromatograms at 280 nm.

## Supplementary Materials

The following are available online at https://www.mdpi.com/2079-6382/9/8/459/s1, Figure S1: (A) HPLC-DAD chromatogram of a methanol extract of the stem bark of Combretum psidioides. Rt 7.4, ellagitannin; Rt 7.9, ellagitannin; Rt 8.3, Sanguiin H-4; Rt 10.159, di-galloyl-glucose; Rt 10.9, ellagitannin; Rt 12.4, corilagin; Rt 15.2, punicalagin; Rt 18.0, ellagic acid derivative; Rt 20.2, 3′-*O*-methyl-4-*O*-(β-d-xylopyranosyl); Rt 25.6, ellagitannin; Rt 29.447, ellagitannin; Rt 30.9, ellagitannin; Rt 32.4, ellagitannin; Rt 33.0, ellagitannin; Rt 35.5, ellagitannin; Rt 38.8, hexagalloylglucose; (B) HPLC-DAD chromatogram of a butanol extract of the stem bark of Combretum psidioides. Rt 7.7, ellagitannin; Rt 8.3, Sanguiin H-4; Rt 10.022, di-galloyl-glucose; Rt 10.776, ellagitannin; Rt 12.244, corilagin; Rt 14.966, punicalagin; Rt 17.565, ellagic acid derivative; Rt 19.846, 3′-*O*-methyl-4-*O*-(β-d-xylopyranosyl); Rt 24.922, ellagic acid derivative; Rt 28.605, ellagitannin; Rt 31.560, ellagitannin; Rt 34.6, ellagitannin; Rt 38.299, hexagalloylglucose. Figure S2: (A) HPLC-DAD chromatograms a methanol extract of the stem bark of Combretum padoides. Rt 7.4, ellagitannin; Rt 8.45, ellagitannin; Rt 9.765, ellagitannin; Rt 10.898, α-Punicalagin anomer or Punicacortein D; Rt 11.69, ellagitannin; Rt 12.437, ellagitannin; Rt 15.246, ellagitannin; Rt 20.23, ellagitannin; Rt 20.447, Ellagic acid arabinoside; Rt 21.4, 3′-*O*-methyl-4-*O*-(β-d-xylopyranosyl) ellagic acid; Rt 29.435, ellagitannin; Rt 32.427, ellagitannin; Rt 35.560, ellagitannin; (B) HPLC-DAD chromatograms a butanol extract of the stem bark of Combretum padoides. Rt 7.4, ellagitannin; Rt 8.45, ellagitannin; Rt 9.768, ellagitannin; Rt 10.903, α-Punicalagin anomer or Punicacortein D; Rt 11.7, ellagitannin; Rt 12.451, ellagitannin; Rt 15.263, ellagitannin; Rt 20.22, ellagitannin; Rt 20.40, Ellagic acid arabinoside; Rt 21.4, 3′-*O*-methyl-4-*O*-(β-d-xylopyranosyl) ellagic acid; Rt 29.4, ellagitannin; Rt 32.4, ellagitannin; Rt 35.5, ellagitannin. Figure S3: (A) HPLC-DAD chromatogram of a butanol extract of the stem bark of Combretum zeyheri. Rt 8.475 min, corilagin derivative; Rt 10.904, ellagitannin; Rt 12.458, ellagitannin; Rt 15.259, gallotannin; Rt 15.66, punicalagin; Rt 17.630, ellagitannin; Rt 20.554, ellagic acid derivative; Rt 24.066, ellagic acid derivative; Rt 26.641, ellagitannin; Rt 29.9, methyl-ellagic acid xyloside; (B) HPLC-DAD chromatogram of a water-soluble extract of the stem bark of Combretum zeyheri. Rt 8.475 min, corilagin derivative; Rt 10.904, ellagitannin; Rt 12.458, ellagitannin; Rt 15.266, gallotannin; Rt 15.671, punicalagin; Rt 17.638, ellagitannin; Rt 20.6, ellagic acid derivative; Rt 24.051, ellagic acid derivative; Rt 26.661, ellagitannin; Rt 29.275, methyl-ellagic acid xyloside. The water-soluble extract contains a high concentration of procyanidines, forming the bump shape of the HPLC-chromatogram.
