# Supplementary material for: HPLC-DAD and UHPLC/QTOF-MS Analysis of Polyphenols in Extracts of the African Species Combretum padoides, C. zeyheri and C. psidioides Related to Their Antimycobacterial Activity
Source: Antibiotics (Basel). 2020 Jul 29;9(8):459. doi: 10.3390/antibiotics9080459 (PMC7460068; doi:10.3390/antibiotics9080459)
Supplement: Supplementary file 1 [file antibiotics-09-00459-s001.zip › Figure 1S.pdf]

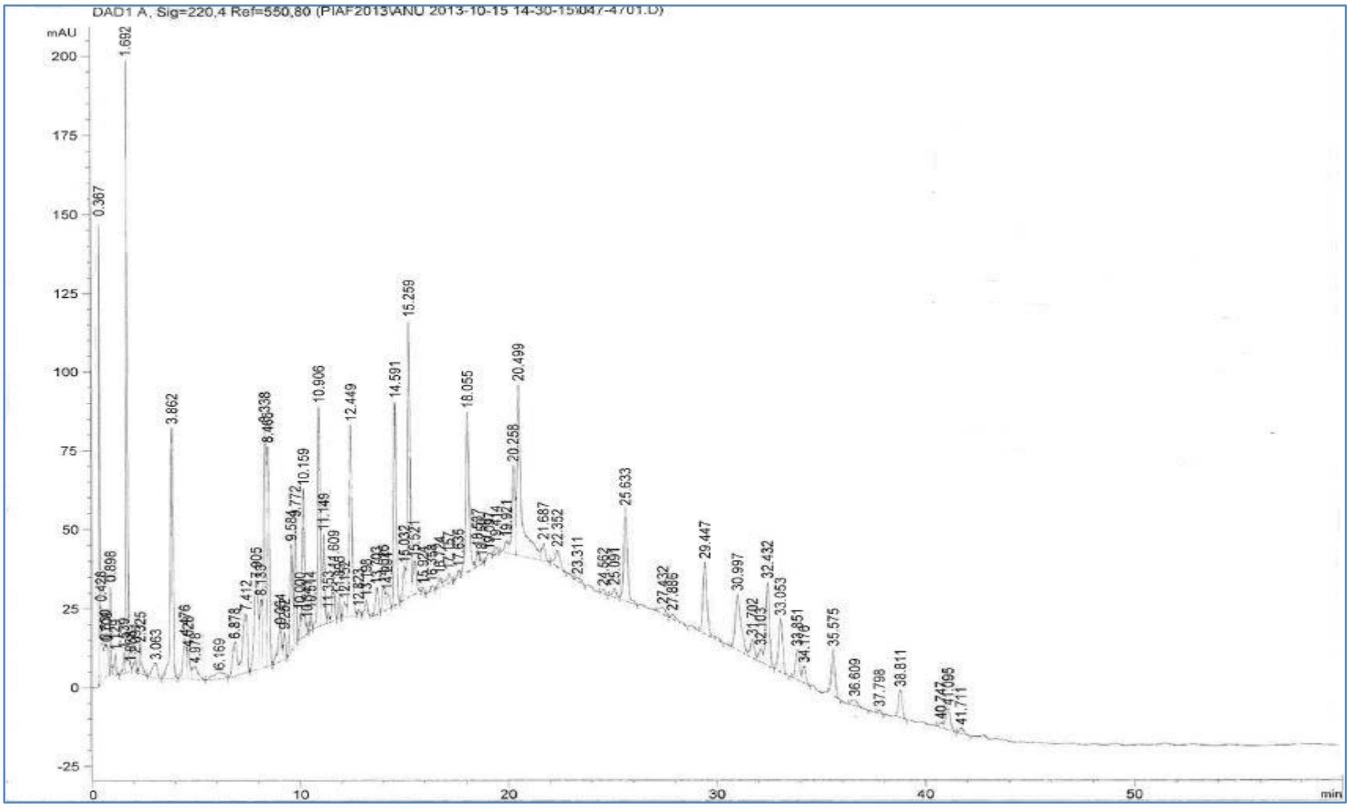

Figure 1S (A). HPLC-DAD chromatogram of a methanol extract of the stem bark of *Combretum psidioides*. Rt 7.4, ellagitannin; Rt 7.9, ellagitannin; Rt 8.3, Sanguiin H-4; Rt 10.159, di-galloyl-glucose; Rt 10.9, ellagitannin; Rt 12.4, corilagin; Rt 15.2, punicalagin; Rt 18.0, ellagic acid derivative; Rt 20.2, 3'-*O*-methyl-4-*O*-(β-D-xylopyranosyl); Rt 25.6, ellagitannin; Rt 29.447, ellagitannin; Rt 30.9, ellagitannin; Rt 32.4, ellagitannin; Rt 33.0, ellagitannin; Rt 35.5, ellagitannin; Rt 38.8, hexagalloylglucose.

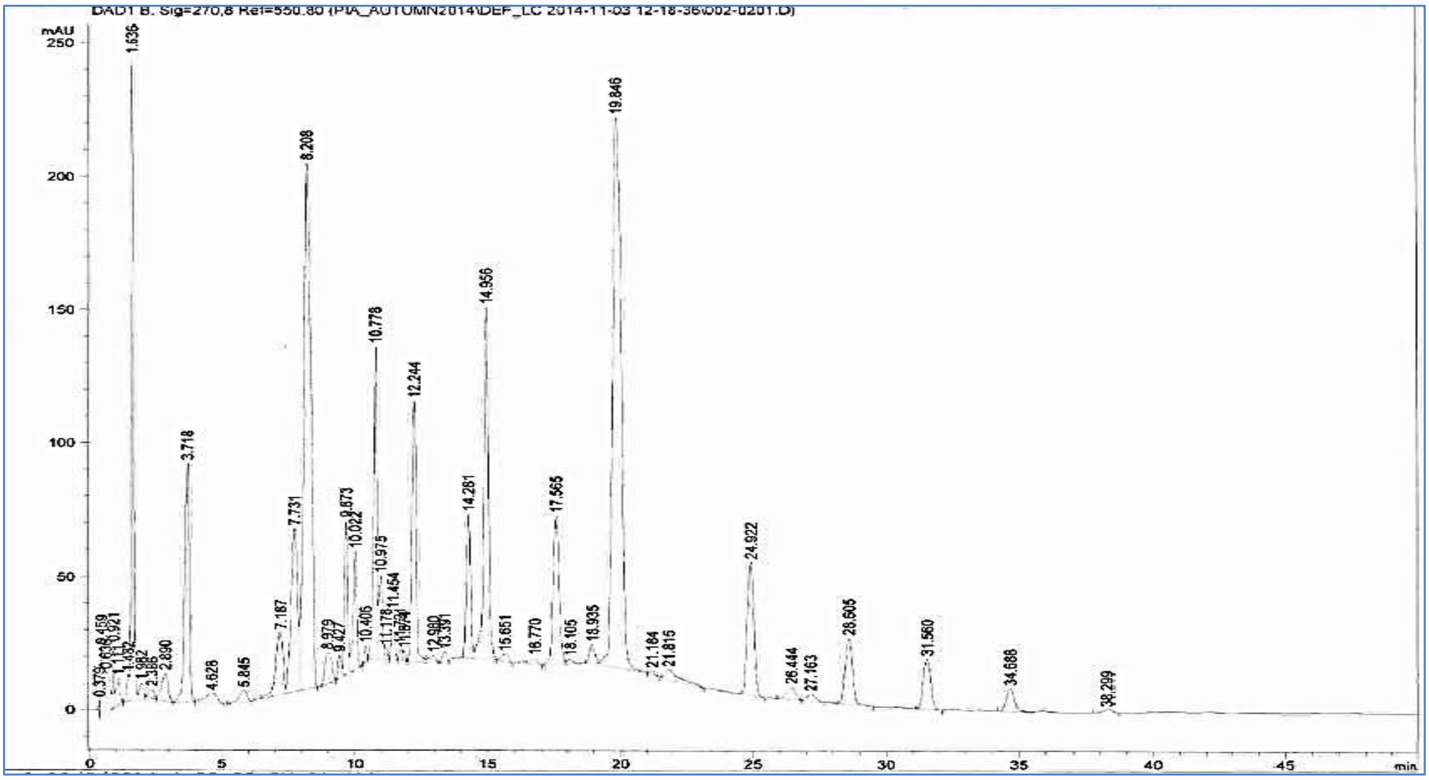

Figure 1S (B). HPLC-DAD chromatogram of a butanol extract of the stem bark of *Combretum psidioides*. Rt 7.7, ellagitannin; Rt 8.3, Sanguiin H-4; Rt 10.022, di-galloyl-glucose; Rt 10.776, ellagitannin; Rt 12.244, corilagin; Rt 14.966, punicalagin; Rt 17.565, ellagic acid derivative; Rt 19.846, 3'-*O*-methyl-4-*O*-(β-D-xylopyranosyl); Rt 24.922, ellagic acid derivative; Rt 28.605, ellagitannin; Rt 31.560, ellagitannin; Rt 34.6, ellagitannin; Rt 38.299, hexagalloylglucose.
