# Supplementary material for: HPLC-DAD and UHPLC/QTOF-MS Analysis of Polyphenols in Extracts of the African Species Combretum padoides, C. zeyheri and C. psidioides Related to Their Antimycobacterial Activity
Source: Antibiotics (Basel). 2020 Jul 29;9(8):459. doi: 10.3390/antibiotics9080459 (PMC7460068; doi:10.3390/antibiotics9080459)
Supplement: Supplementary file 1 [file antibiotics-09-00459-s001.zip › Figure 2S.pdf]

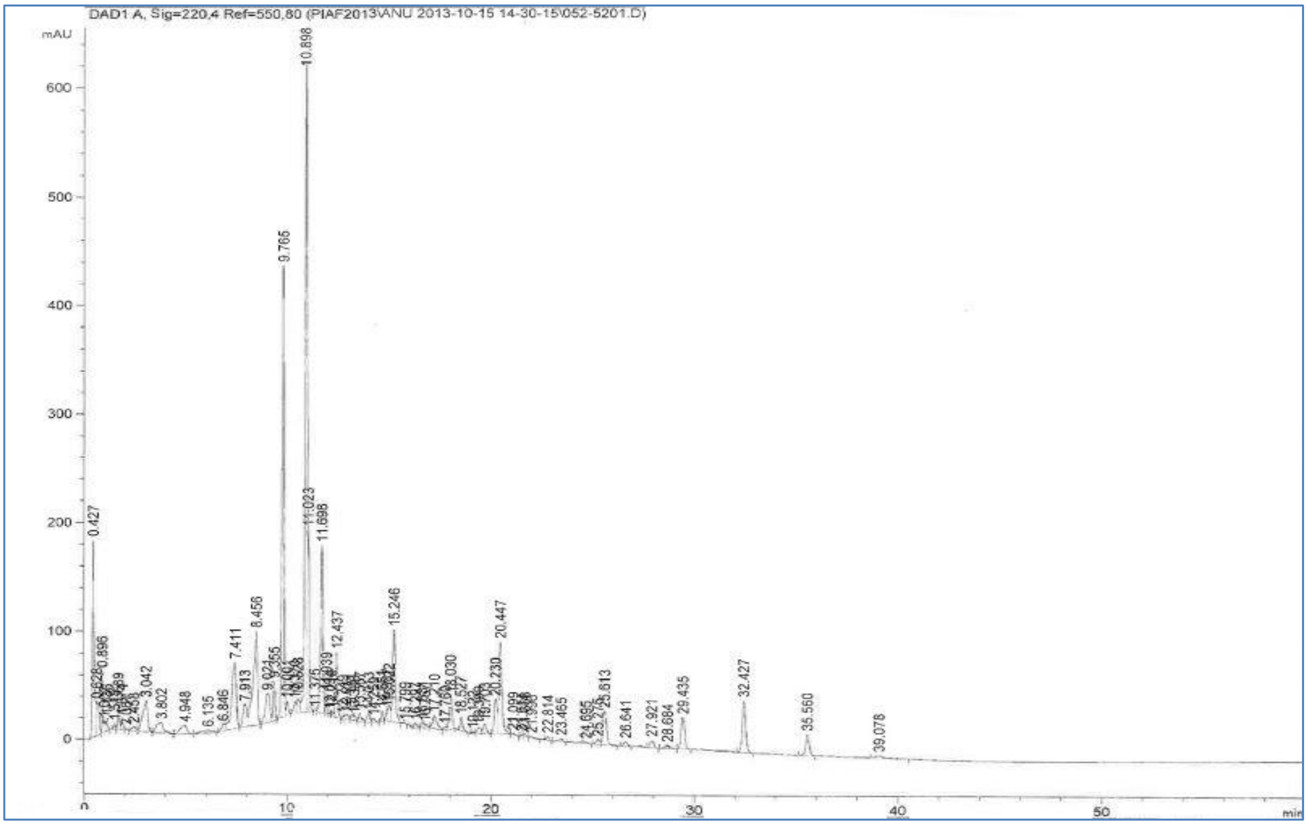

Figure 2S (A). HPLC-DAD chromatograms a methanol extract of the stem bark of *Combretum padoides.* Rt 7.4, ellagitannin; Rt 8.45, ellagitannin; Rt 9.765, ellagitannin; Rt 10.898, α-Punicalagin anomer or Punicacortein D; Rt 11.69, ellagitannin; Rt 12.437, ellagitannin; Rt 15.246, ellagitannin; Rt 20.23, ellagitannin; Rt 20.447, Ellagic acid arabinoside; Rt 21.4, 3'-*O*-methyl-4-*O*-(β-D-xylopyranosyl) ellagic acid; Rt 29.435, ellagitannin; Rt 32.427, ellagitannin; Rt 35.560, ellagitannin.

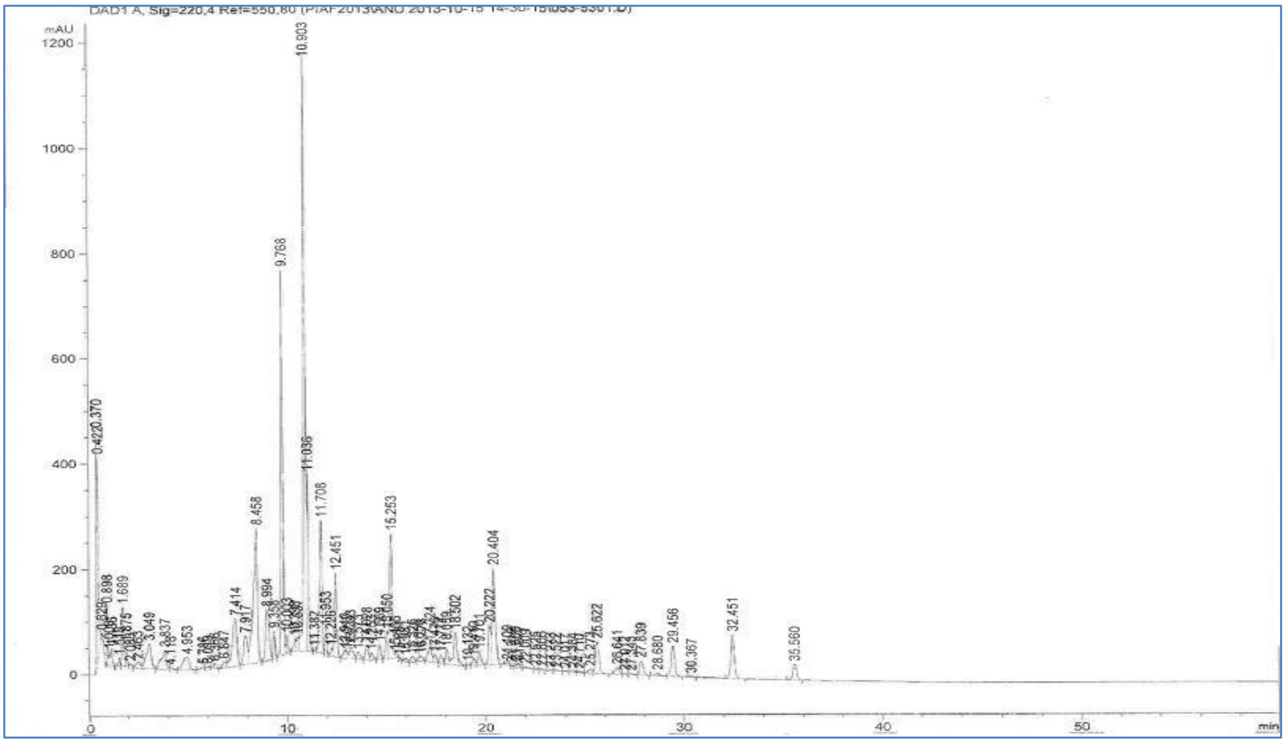

Figure 2S (B). HPLC-DAD chromatograms a butanol extract of the stem bark of *Combretum padoides.* Rt 7.4, ellagitannin; Rt 8.45, ellagitannin; Rt 9.768, ellagitannin; Rt 10.903, α-Punicalagin anomer or Punicacortein D; Rt 11.7, ellagitannin; Rt 12.451, ellagitannin; Rt 15.263, ellagitannin; Rt 20.22, ellagitannin; Rt 20.40, Ellagic acid arabinoside; Rt 21.4, 3'-*O*-methyl-4-*O*-(β-D-xylopyranosyl) ellagic acid; Rt 29.4, ellagitannin; Rt 32.4, ellagitannin; Rt 35.5, ellagitannin.
