# Supplementary material for: HPLC-DAD and UHPLC/QTOF-MS Analysis of Polyphenols in Extracts of the African Species Combretum padoides, C. zeyheri and C. psidioides Related to Their Antimycobacterial Activity
Source: Antibiotics (Basel). 2020 Jul 29;9(8):459. doi: 10.3390/antibiotics9080459 (PMC7460068; doi:10.3390/antibiotics9080459)
Supplement: Supplementary file 1 [file antibiotics-09-00459-s001.zip › Figure 3S.pdf]

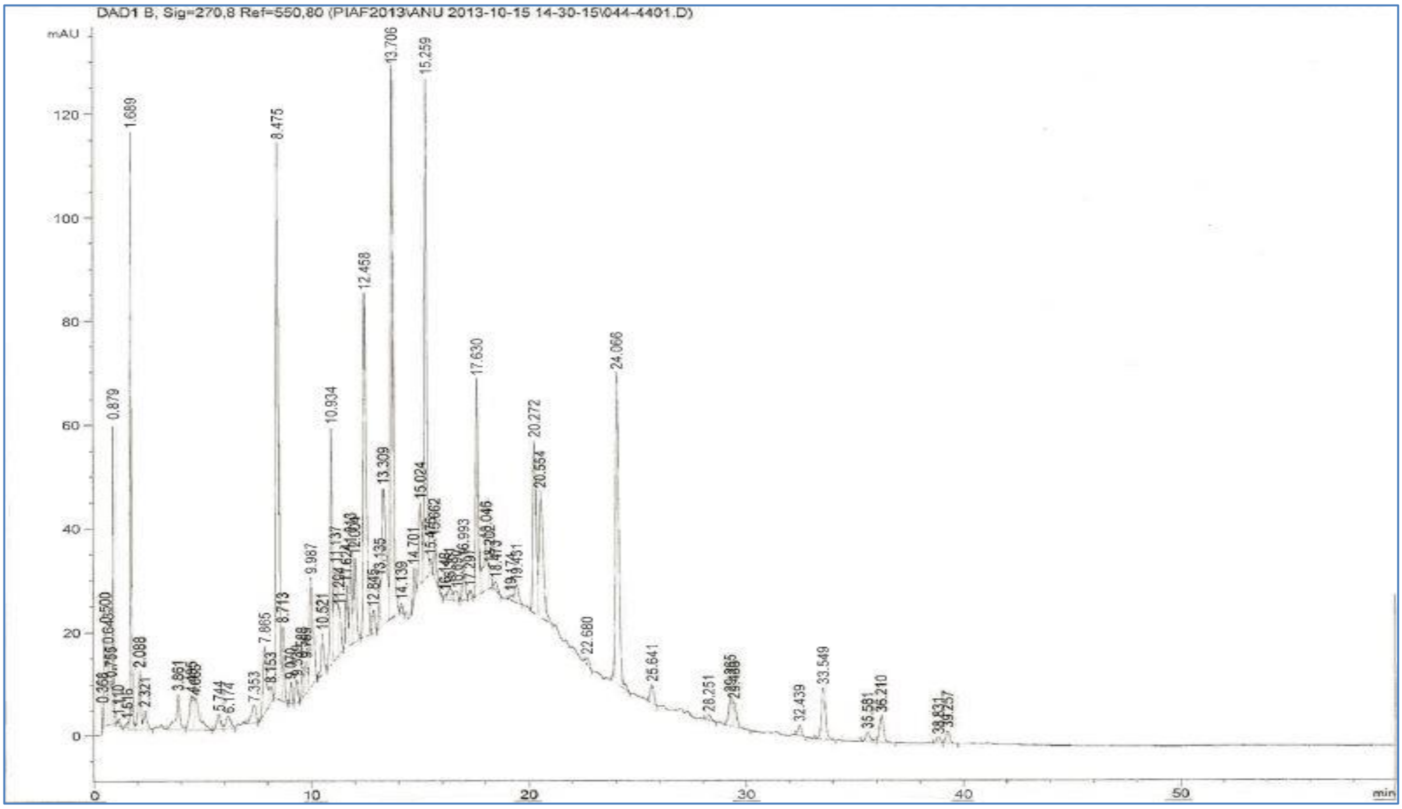

Figure 3S (A). HPLC-DAD chromatogram of a butanol extract of the stem bark of *Combretum zeyheri*. Rt 8.475 min, corilagin derivative; Rt 10.904, ellagitannin; Rt 12.458, ellagitannin; Rt 15.259, gallotannin; Rt 15.66, punicalagin; Rt 17.630, ellagitannin; Rt 20.554, ellagic acid derivative; Rt 24.066, ellagic acid derivative; Rt 26.641, ellagitannin; Rt 29.9, methyl-ellagic acid xyloside.

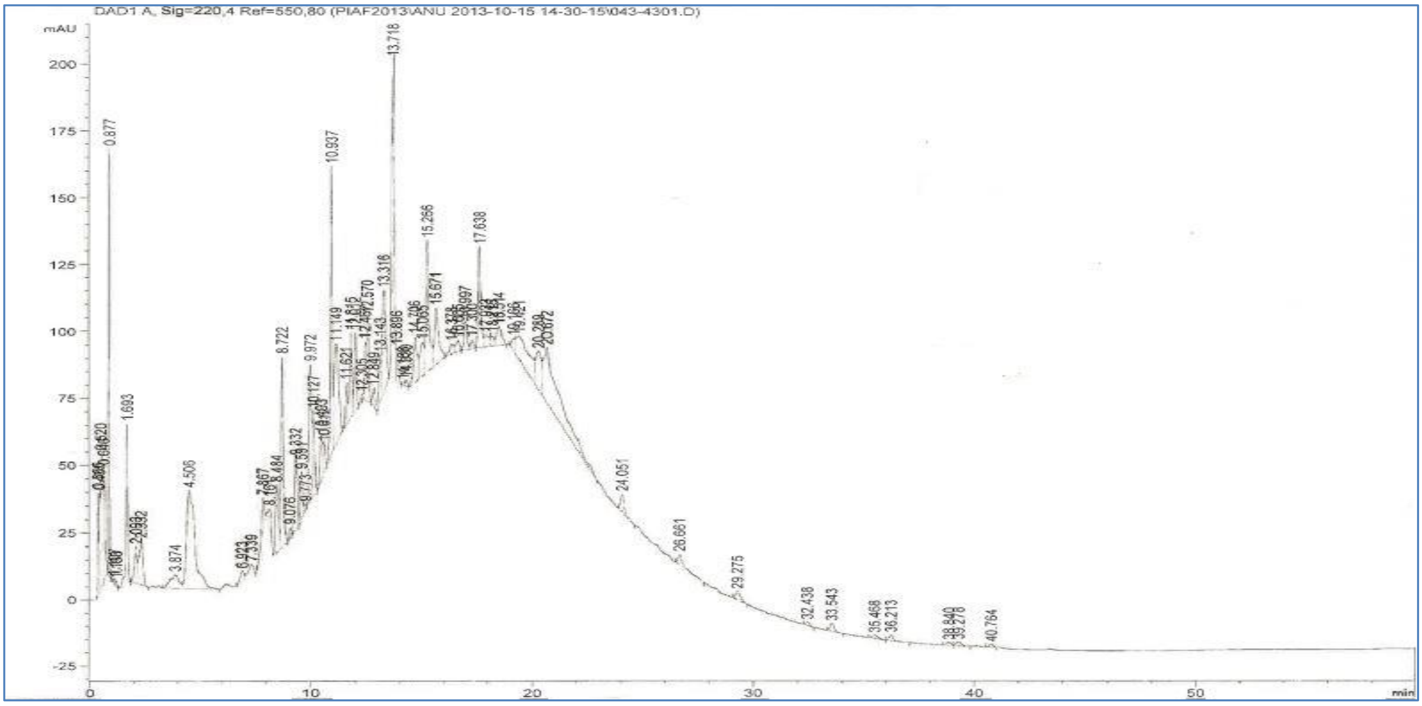

Figure 3S (B). HPLC-DAD chromatogram of a water-soluble extract of the stem bark of *Combretum zeyheri*. Rt 8.475 min, corilagin derivative; Rt 10.904, ellagitannin; Rt 12.458, ellagitannin; Rt 15.266, gallotannin; Rt 15.671, punicalagin; Rt 17.638, ellagitannin; Rt 20.6, ellagic acid derivative; Rt 24.051, ellagic acid derivative; Rt 26.661, ellagitannin; Rt 29.275, methyl-ellagic acid xyloside. The water-soluble extract contains a high concentration of procyanidines, forming the bump shape of the HPLC-chromatogram.
